# Supplementary material for: Injections of Predatory Bacteria Work Alongside Host Immune Cells to Treat Shigella Infection in Zebrafish Larvae
Source: Curr Biol. 2016 Dec 19;26(24):3343–51. doi: 10.1016/j.cub.2016.09.067 (PMC5196024; doi:10.1016/j.cub.2016.09.067)
Supplement: Document S1. Supplemental Experimental Procedures and Figures S1–S3 [file mmc1.pdf]

Current Biology, Volume *26*

Supplemental Information

Injections of Predatory Bacteria

Work Alongside Host Immune Cells

to Treat *Shigella* Infection in Zebrafish Larvae

Alexandra R. Willis, Christopher Moore, Maria Mazon-Moya, Sina Krokowski, Carey Lambert, Robert Till, Serge Mostowy, and R. Elizabeth Sockett

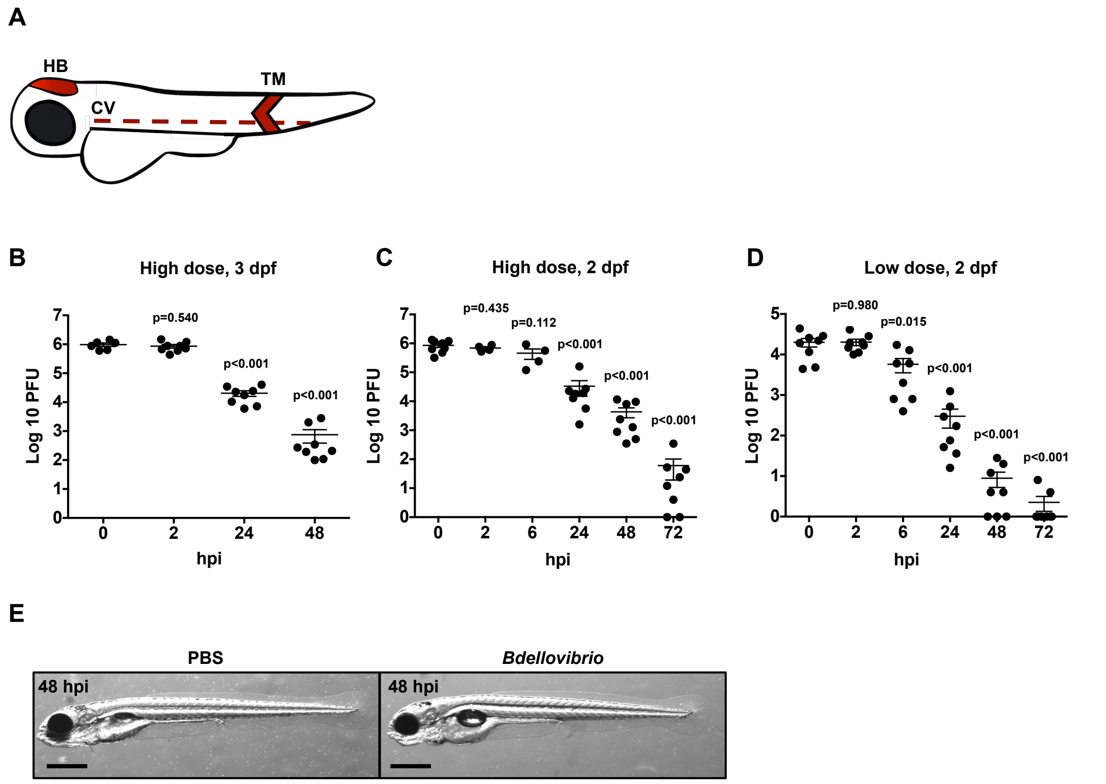

Figure S1. Injected Predatory *Bdellovibrio* Persist in Zebrafish Larvae and are Ultimately Cleared, Related to Figure 1

(A) Zebrafish larvae were injected with bacteria or PBS in either the hindbrain ventricle (HB), tail muscle (TM) or caudal vein (CV) shaded in red. Injections in the hindbrain were performed to study population level dynamics of *Bdellovibrio*, *Shigella* and/or leukocytes. Injections in the tail muscle were performed to enable high-resolution confocal microscopy.

(B–D) WT AB zebrafish larvae were injected with (B-C) a high dose of *Bdellovibrio* or (D) a low dose of *Bdellovibrio* at (B) 3 dpf or (C-D) 2 dpf in the hindbrain ventricle. Live *Bdellovibrio* were enumerated from larval homogenates at time points indicated. Each circle represents a count from an individual larva. Mean ± SEM (horizontal bars) are shown. (B) Data pooled from 2 independent experiments using 3-4 larvae per time point. (C) Data from 2 independent experiments using 4 larvae per time point. p values (versus the 0 hpi time point) determined by multiple t-test. Significance with Bonferroni correction defined as $p<0.017$ for (B), $p<0.01$ for (C-D).

(E) WT AB zebrafish larvae were injected in the hindbrain at 3 dpf with PBS or 1-2 x 10^5 PFU of *Bdellovibrio* and imaged via stereomicroscopy at 48 hpi (i.e. 5 dpf). Representative images of larval morphology are shown. Scale bar = 0.5 mm.

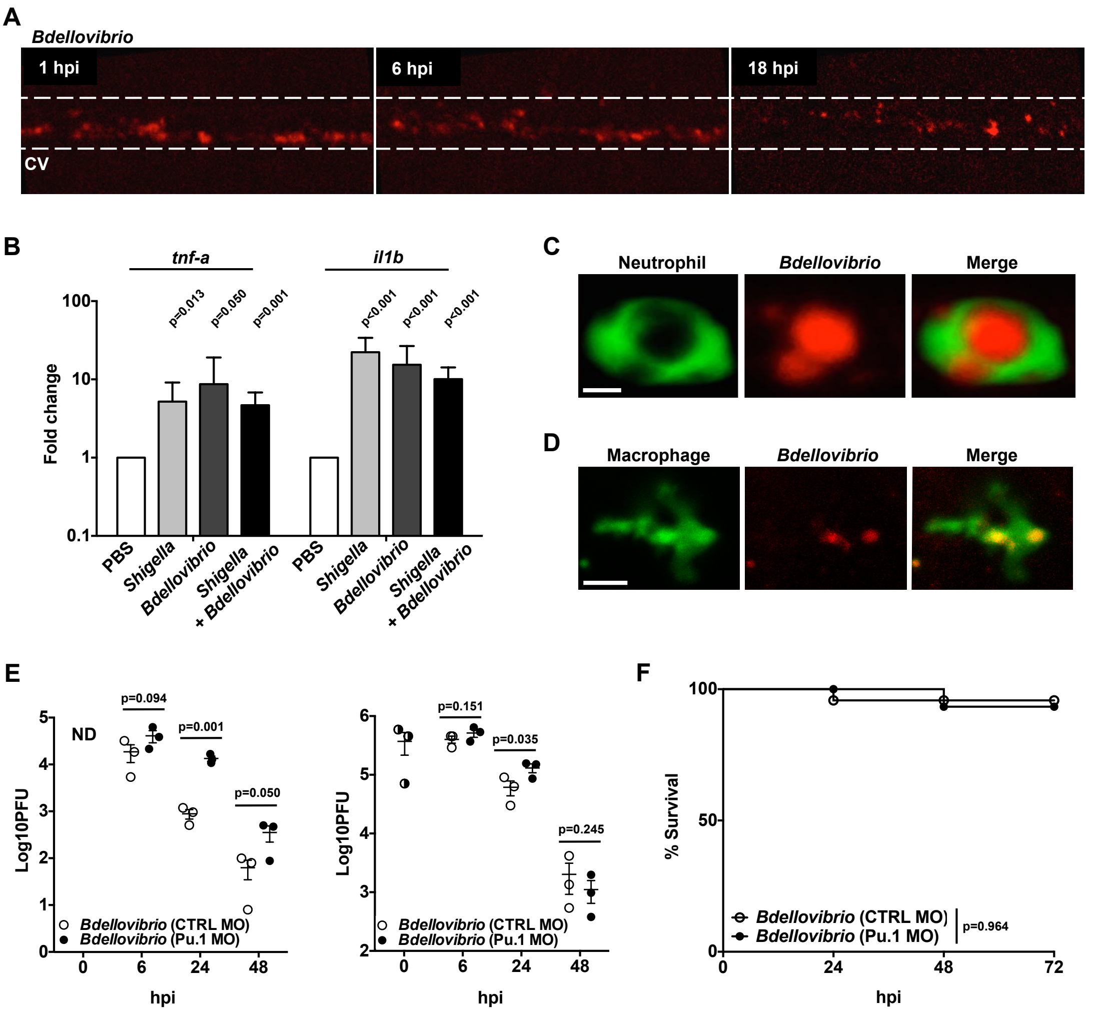

Figure S2. Characterisation of the Innate Immune Response to *Bdellovibrio* In Vivo, Related to Figure 3

(A) 1-2 x 10^5 PFU of mCherry-*Bdellovibrio* (red) were injected into the caudal vein of WT AB zebrafish larvae at 3 dpf and imaged at the stereomicroscope. The same larva was imaged over time. The area inside the white dashed line indicates the blood stream. CV, caudal vein.

(B) WT zebrafish larvae were injected with: PBS, >5 x 10^3 CFU of *S. flexneri*, 1-2 x10^5 PFU of *Bdellovibrio* or co-injected with the above doses of *S. flexneri* and *Bdellovibrio*, sequentially. RNA was extracted from pools of 5 larvae and (2 technical replicates for each of 3 biological repeats for *tnf-a*, 2 technical replicates for each of 4 biological repeats for *il1b*; normalised to PBS). Expression of *il1b* and *tnf-a* mRNA

transcripts was determined by qRT-PCR. p values (versus PBS control) determined by unpaired 1-tailed Student's t-test. Significance defined as $p<0.05$.

(C) 1-2 x 10^5 PFU of mCherry-*Bdellovibrio* were injected into the tail muscle of Tg(*mpx*:GFP)^{i114} zebrafish larvae at 3 dpf and single-cell interactions between *Bdellovibrio* (red) and neutrophils (green) visualised by high-resolution confocal microscopy at 100x magnification. A representative image is shown. Scale bar = 5 µm. See also Movie S4.

(D) 1-2 x 10^5 PFU of mTeal-*Bdellovibrio* were injected into the tail muscle of Tg(*mpeg1*:Gal4-FF)^{gl25}/Tg(UAS-E1b:nfsB.mCherry)^{c264} zebrafish larvae at 3 dpf and single-cell interactions between *Bdellovibrio* (red) and macrophages (green) visualised by high-resolution confocal microscopy at 63x magnification. A representative image is shown. Scale bar = 10 µm. See also Movie S4.

(E) Tg(*mpx*:GFP)^{i114} zebrafish larvae were pre-treated using control (CTRL) or Pu.1-targeting morpholino (MO) to deplete leukocytes. Morphants were injected at 3 dpf with either PBS or 0.1-6 x 10^5 PFU of mCherry-*Bdellovibrio*. Live *Bdellovibrio* were enumerated from PBS homogenates of larvae. Each circle represents a count from an individual larva. Half-filled circles represent enumerations from larvae at time 0 and are representative of inocula for both conditions. Mean ± SEM (horizontal bars) are shown. p values (between conditions at cognate timepoints) determined by multiple t-test. Significance with Bonferroni correction defined as $p<0.017$. As inocula from independent experiments were variable up to a log-fold (PFU counting of predators necessarily performed post experiment), representatives of 3 independent experiments performed are shown. See also Figure 3E.

(F) Survival curve of Tg(*mpx*:GFP)^{i114} zebrafish larvae injected with control (CTRL) or Pu.1-targeting morpholino (MO) and then injected at 3 dpf with either PBS or 1-2 x 10^5 PFU of mCherry-*Bdellovibrio*. Larvae were incubated at 28°C for 72 hpi. Data pooled from 2 independent experiments using n = 21-26 larvae per treatment per experiment. Up to 3 larvae per condition were taken for CFU at 24 and 48 hr

timepoints. Mean ± SEM (horizontal bars) are shown. p value between conditions determined by log rank Mantel-Cox test. Significance defined as $p<0.05$.

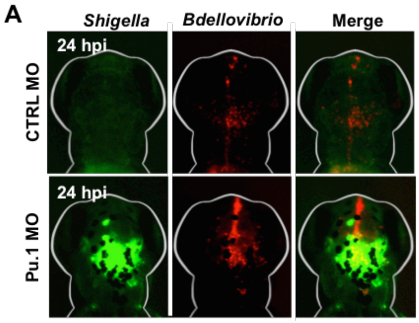

Figure S3. ***Bdellovibrio*** **Work Alongside Innate Immune Cells to Protect Against** ***Shigella*** **Infection In Vivo, Related to Figure 4**

(A) WT AB zebrafish larvae were injected with control (CTRL) or Pu.1-targeting morpholino (MO). Morphants were injected at 3 dpf in the hindbrain ventricle with > 5 x 10^3 of GFP-*S. flexneri* and treated with injection of 1-2 x 10^5 PFU of mCherry-*Bdellovibrio* 30-90 min after initial injection. Representative images of bacteria in the hindbrain ventricle in CTRL or Pu.1 morphants are shown.

SUPPLEMENTAL EXPERIMENTAL PROCEDURES

Ethics Statement

Animal experiments conducted at Imperial College London and The University of Nottingham were performed according to the Animals (Scientific Procedures) Act 1986, and were approved by the Home Office under Project Licenses: PPL 70/7446 and PPL 30/3378, respectively.

Zebrafish Husbandry

Wild type AB zebrafish were purchased from the Zebrafish International Resource Center (Eugene, OR). Tg*(mpx:GFP)*^{i114} and Tg(*mpeg1*:Gal4-FF)^{gl25}/Tg(UAS-E1b:nfsB.mCherry)^{c264} transgenic zebrafish lines are previously described [S1, S2]. Zebrafish embryos were obtained from natural spawning, bleached and raised at 28°C in E2 medium [S3]. Embryos reared for the purpose of microscopy were supplemented with 0.003% 1-phenyl-2-thiourea (Sigma-Aldrich) from 24 hours post fertilisation to prevent melanin synthesis. Zebrafish were developmentally staged according to Kimmel et al [S4]. Where specified, larvae were anaesthetised with 200 µg/ml Tricaine (Sigma-Aldrich).

Bacterial Preparations

Bacterial strains are described in the Table below. For experiments shown in Figures 1E-G, 2A, 2E-2F, 3A and S2A, non-chromosomal, cytoplasmically fluorescent *B. bacteriovorus* HD100:pK18 Bd0064mCherry (mCherry-*Bdellovibrio*) and *B. bacteriovorus* HD100:pK18 Bd0064mTeal (mTeal-*Bdellovibrio*) were prepared by predation on *E. coli*:pZMR100 prey in CaHEPES buffer (25 mM HEPES, 2 mM $CaCl_2$, pH 7.6) in the presence of 25 µg/ml kanamycin. *B. bacteriovorus* were filtered through a 0.45 µm filter to remove residual *E. coli*, washed twice with phosphate-buffered saline (PBS) to remove residual kanamycin (if present) and resuspended in PBS at 25°C. Fluorescence was eventually lost for non-chromosomally tagged

strains upon predatory replication in the absence of kanamycin selection inside zebrafish larvae. Permanently fluorescent *B. bacteriovorus* strains were constructed with chromosomal integration of Bd0064mCherry or Bd0064mTeal at the wild type gene locus and were cultured as above but in the absence of kanamycin. Permanently-tagged strains were used in all other experiments. *Bdellovibrio* suspensions were observed microscopically for purity and motility and concentrated immediately prior to injection via centrifugation. As enumerations of *Bdellovibrio* are not possible by optical density, standardised culturing was employed to give reproducible predator numbers and retrospective live cell counting of each *Bdellovibrio* culture was achieved by plaque assay counts as described in section below 'Quantification of Bacterial Burden'. Therefore, as enumeration was only available after the experiment, a small range of *Bdellovibrio* numbers may be quoted as the employed PFU.

Streptomycin- and carbenicillin- resistant *S. flexneri* serotype 5a M90T expressing green fluorescent protein (GFP-*Shigella*) was cultured overnight in trypticase soy broth (TSB) at 37°C in the presence of 50 µg/ml carbenicillin. Cultures were diluted 32 x in fresh TSB and grown until OD_{600nm} = 0.6. Bacteria were harvested via centrifugation, for 5 min at 4200 rpm, washed twice in PBS to remove carbenicillin and reconstituted in PBS.

Table. Bacterial Strains Used in This Study

Bacterial strain	Notes	Source
Shigella flexneri serotype 5a M90T-GFP	CbR, SmR	[S5]
Bdellovibrio bacteriovorus HD100:pK18 Bd0064mTeal	KanR	This work
Bdellovibrio bacteriovorus HD100:pK18 Bd0064mCherry	KanR	This work
Bdellovibrio bacteriovorus HD100 Bd0064mCherry-chromosomal	-	This work
Bdellovibrio bacteriovorus HD100 Bd0064mTeal-chromosomal	-	This work
Escherichia coli:S17-1 pZMR100	KanR	[S6]
Escherichia coli S17-1	-	[S7]

CbR, carbenicillin resistance; SmR, streptomycin resistance; KanR, kanamycin resistance

In Vitro Predation Assays

Viable counts, microscopic plate reader assays and microscopic observation of predation were carried out on *Bdellovibrio* and *Shigella* mixtures plus controls in CaHEPES buffer. For viable counting, 6.2 x 10^{10} PFU of 0.45 µm- filtered predator *bd0064*mCherry-*Bdellovibrio* and 5-12 x 10^8 CFU of prey GFP-*Shigella* (prepared as above) were co-incubated in 10 ml CaHEPES buffer at 29°C and enumerated over time (Figure 2B). Predation from such bacterial cultures in CaHEPES was visualised via wide field microscopy (Figure 2A). Enumeration was by CFU plating for *Shigella* on Luria-Bertani (LB) agar. Enumeration of *Bdellovibrio* inoculum was by PFU plating on *E.coli* lawns on YPSC plates, as described below. For plate reader assays, 2-7 x 10^7 CFU of GFP-*Shigella* and 8.4-10.4 x 10^9 PFU of mCherry-*Bdellovibrio* were mixed in 200 µl CaHEPES buffer. OD_{600} and mCherry fluorescence were measured every 30 min for 6 hr at 37°C using a microplate reader (TECAN Infinite M200 Pro) but for simplicity counts were displayed every hr (Figures 2C-2D).

Morpholino Injection of Zebrafish Embryos

Antisense morpholino oligonucleotides were purchased from GeneTools (www.gene-tools.com). For leukocyte depletion experiments, published morpholinos were used to target zebrafish Pu.1, as previously described [S5, S8, S9]. To control for the effects of morpholino injection, a morpholino with no known target in the zebrafish genome was also used [S5]. Injections were performed into 1-8 cell stage embryos. Morpholinos were diluted in 0.1% phenol red (Sigma) to concentrations of 0.5-1 mM and 1-4 nl administered per embryo. Doses used were chosen to maximise leukocyte knockdown while minimising larval toxicity. Efficacy of Pu.1 morpholinos was confirmed in both Tg*(mpx:GFP)*^{i114} and Tg(*mpeg1*:Gal4-FF)^{gl25}/Tg(UAS-E1b:nfsB.mCherry)^{c26} transgenic zebrafish larvae. Larvae screened before use showed normal morphology and locomotion, an absence of macrophages and a ~50% reduction in neutrophils (typically from ~160 to ~80).

Zebrafish Infections

For hindbrain infection assays, 1-2 nl of bacterial suspension i.e. 1-2 x 10^5 PFU of *Bdellovibrio* or >5 x 10^3 CFU of *Shigella*, or PBS were delivered to the hindbrain ventricle of anaesthetised WT AB zebrafish larvae at 3 dpf via microinjection. To image single-cell interactions via confocal microscopy, tail muscle infections were performed to enable precise high-resolution imaging (Figures 2E-2F). Here, the tail muscles of zebrafish larvae were injected with 10^3 CFU of *Shigella* and 1-2 x 10^5 PFU of *Bdellovibrio*. For *Bdellovibrio* replication assays (Figure 2G), an altered ratio of predator: prey was used (i.e. 2-6 x 10^5 CFU of *Shigella* and 1-30 x 10^2 PFU of *Bdellovibrio*) to allow detection of progeny *Bdellovibrio* emerging from *Shigella* prey after replication, without being masked by a large standing number of non-invading *Bdellovibrio*. For all coinfection assays, *Bdellovibrio* was injected 30-90 min post *Shigella* infection. To test interactions with neutrophils, we used Tg*(mpx:GFP)*^{i114}

larvae with GFP-neutrophils. To study macrophages, we used Tg(*mpeg1*:Gal4-FF)^{gl25}/Tg(UAS-E1b:nfsB.mCherry)^{c26} larvae with mCherry-expressing macrophages. With the exception of Figures 2E-2F, S2A and S2C-D, all experiments including survival assays, bacterial enumerations and population level imaging via stereomicroscopy, injections were performed in the hindbrain ventricle.

Quantification of Bacterial Burden

To determine *Shigella* inoculum or burden at later timepoints, larvae were sacrificed with Tricaine, washed twice in PBS-Tricaine, lysed in 200 µl of PBS 0.1% Triton X-100 and homogenised mechanically for recovery of host-invasive *Shigella* pathogens from within the larval tissue [S5]. Here, larval homogenates were serially diluted in PBS and plated on LB agar supplemented with 50 µg/ml carbenicillin. Plates were incubated at 37°C and GFP-expressing CFU enumerated. Only viable larvae were included in our analyses.

To determine *Bdellovibrio* inoculum or burden at later timepoints, larval homogenates were prepared as described for *Shigella* above but instead homogenising in 200 µl of PBS alone as *Bdellovibrio*, unlike *Shigella*, are susceptible to Triton. PBS-treated homogenates were serially diluted and plated onto yeast extract-peptone-calcium (YPSC) soft agar overlay plates with *E. coli* S17-1 provided in the top agar to produce prey lawns for plaque formation by each individual *Bdellovibrio* [S10]. Plates were incubated for 5-10 days at 30ºC and *Bdellovibrio* enumerated as PFU.

For the co-injected *Shigella* plus *Bdellovibrio,* larval homogenates were prepared in PBS 0.1% Triton X-100. *Bdellovibrio* numbers could therefore not be co-determined as they were severely reduced by Triton treatment, required to liberate *Shigella* from host tissues.

Live Imaging, Image Processing and Analysis

For whole-animal in vivo imaging, anaesthetised zebrafish larvae were immobilised in 1% low melting point agarose and submerged in E2 supplemented with Tricaine as previously described [S11]. Visualisation of hindbrain infections was via transmission or fluorescence stereomicroscopy using a Leica M205FA microscope. Multiple position Z-stacks were acquired with a 10x (NA 0.5) dry objective, using 60 mm dishes. High-resolution imaging of tail muscle infections was via confocal microscopy using a Zeiss LSM 710 microscope. Z-stacks were acquired with a 40x, 63x or 100x oil immersion objective and subject to maximum intensity projection, using 35 mm glass-bottom dishes. Imaging was performed at 28°C. .PNG and .AVI files were processed using ImageJ/FIJI software.

Quantitative Reverse Transcription PCR (qRT-PCR) of Zebrafish Cytokines

Total RNA was extracted from pools of 5 bacterially injected or control larvae at 4 hpi using RNAqueous Kit (Ambion) and cDNA obtained using a QuantiTect reverse transcription kit (Qiagen). qRT-PCR was using SYBR green PCR master mix (Applied Biosystems) and performed on a Rotor-GeneQ thermocycler (Qiagen). Published primers were used to measure *tnf-α* and *il1b* [S12]. The housekeeping gene *gapdh* (using primers: FW-5'- TGGGCCAATGAAGGGAATTCTGGGAT-3' and RV-5'- TAACAGGTCAGCAACACGATGGCT-3') was used to normalise quantities of cDNA, using the $2^{-\Delta\Delta CT}$ method [S13]. Quantifications were performed on duplicate wells.

Statistical Analysis

All p values are displayed directly on Figures for clarity. p values for bacterial enumerations, leukocyte quantifications and cytokine data (Figures 1F, 2G, 3C-3E, 4A, S2B and S2E), determined by 1-tailed, unpaired Student's t-test on Prism

software (GraphPad Software Inc.). p values for bacterial enumerations in (Figures 1C, 2G and S1B-S1D) determined by multiple t-test on Prism software. p values for survival and in vitro assays (Figures 1G, 2B-2D, 4B and S2F) determined by log rank Mantel-Cox test on Prism software. Statistical significance is defined as p value <0.05, unless otherwise mentioned (i.e. Bonferroni correction for multiple testing).

SUPPLEMENTAL REFERENCES

S1. Ellett, F., Pase, L., Hayman, J.W., Andrianopoulos, A., and Lieschke, G.J. (2011). *mpeg1* promoter transgenes direct macrophage-lineage expression in zebrafish. Blood *117*, e49-56.

S2. Gray, C., Loynes, C.A., Whyte, M.K., Crossman, D.C., Renshaw, S.A., and Chico, T.J. (2011). Simultaneous intravital imaging of macrophage and neutrophil behaviour during inflammation using a novel transgenic zebrafish. Thromb Haemost. *105*, 811-819.

S3. Westerfield, M. (1995). The zebrafish book : a guide for the laboratory use of zebrafish (*Danio rerio*), Ed. 3. Edition, (Eugene, OR: M. Westerfield).

S4. Kimmel, C.B., Ballard, W.W., Kimmel, S.R., Ullmann, B., and Schilling, T.F. (1995). Stages of embryonic development of the zebrafish. Developmental dynamics : an official publication of the American Association of Anatomists. *203*, 253-310.

S5. Mostowy, S., Boucontet, L., Mazon Moya, M.J., Sirianni, A., Boudinot, P., Hollinshead, M., Cossart, P., Herbomel, P., Levraud, J.P., and Colucci-Guyon, E. (2013). The zebrafish as a new model for the in vivo study of *Shigella flexneri* interaction with phagocytes and bacterial autophagy. PLoS Pathog. *9*, e1003588.

S6. Rogers, M., Ekaterinaki, N., Nimmo, E., and Sherratt, D. (1986). Analysis of Tn7 transposition. Mol. Gen. Genet. *205*, 550-556.

S7. Simon, R., Priefer, U., and Puhler, A. (1983). A broad host range mobilization system for in vivo genetic-engineering - transposon mutagenesis in Gram-negative bacteria. Bio-Technol. *1*, 784-791.

S8. Clay, H., Davis, J.M., Beery, D., Huttenlocher, A., Lyons, S.E., and Ramakrishnan, L. (2007). Dichotomous role of the macrophage in early

Mycobacterium marinum infection of the zebrafish. Cell Host Microbe *2*, 29-39.

S9. Su, F., Juarez, M.A., Cooke, C.L., Lapointe, L., Shavit, J.A., Yamaoka, J.S., and Lyons, S.E. (2007). Differential regulation of primitive myelopoiesis in the zebrafish by Spi-1/Pu.1 and C/ebp1. Zebrafish *4*, 187-199.

S10. Stolp, H., and Starr, M.P. (1963). *Bdellovibrio bacteriovorus* Gen. Et Sp. N., a predatory, ectoparasitic, and bacteriolytic microorganism. Antonie van Leeuwenhoek *29*, 217-248.

S11. Mazon Moya, M.J., Colucci-Guyon, E., and Mostowy, S. (2014). Use of *Shigella flexneri* to study autophagy-cytoskeleton interactions. J. Vis. Exp. e51601.

S12. Stockhammer, O.W., Zakrzewska, A., Hegedus, Z., Spaink, H.P., and Meijer, A.H. (2009). Transcriptome profiling and functional analyses of the zebrafish embryonic innate immune response to *Salmonella* infection. J. Immunol. *182*, 5641-5653.

S13. Livak, K.J., and Schmittgen, T.D. (2001). Analysis of relative gene expression data using real-time quantitative PCR and the 2(-Delta Delta C(T)) Method. Methods *25*, 402-408.
