# Supplementary figures and images for: Injections of Predatory Bacteria Work Alongside Host Immune Cells to Treat Shigella Infection in Zebrafish Larvae
Source: Curr Biol. 2016 Dec 19;26(24):3343–51. doi: 10.1016/j.cub.2016.09.067 (PMC5196024; doi:10.1016/j.cub.2016.09.067)

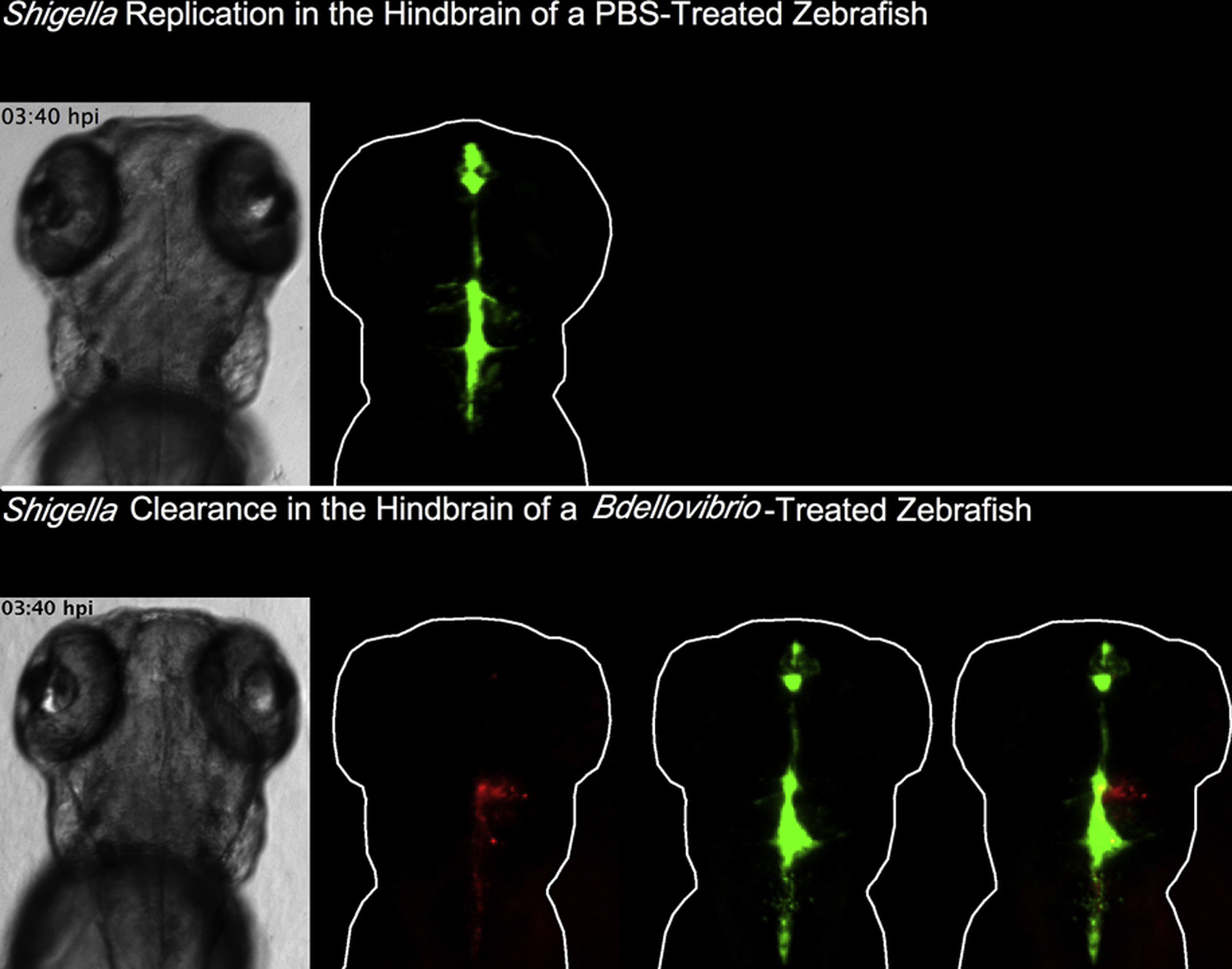

Supplement: Movie S1. Bdellovibrio Is Therapeutic to Shigella Infection In Vivo, Related to Figure 1 — WT AB zebrafish larvae were injected in the hindbrain ventricle with >5 × 10^3^ CFU of GFP-S. flexneri (green) followed by a hindbrain injection of PBS or 1-2× 10^5^ PFU mCherry-Bdellovibrio (red) 30-90 min after initial Shigella infection. The infection process was visualized by fluorescent stereomicroscopy. Z stacks were acquired at 20 or 5 min intervals. [file mmc2.jpg]

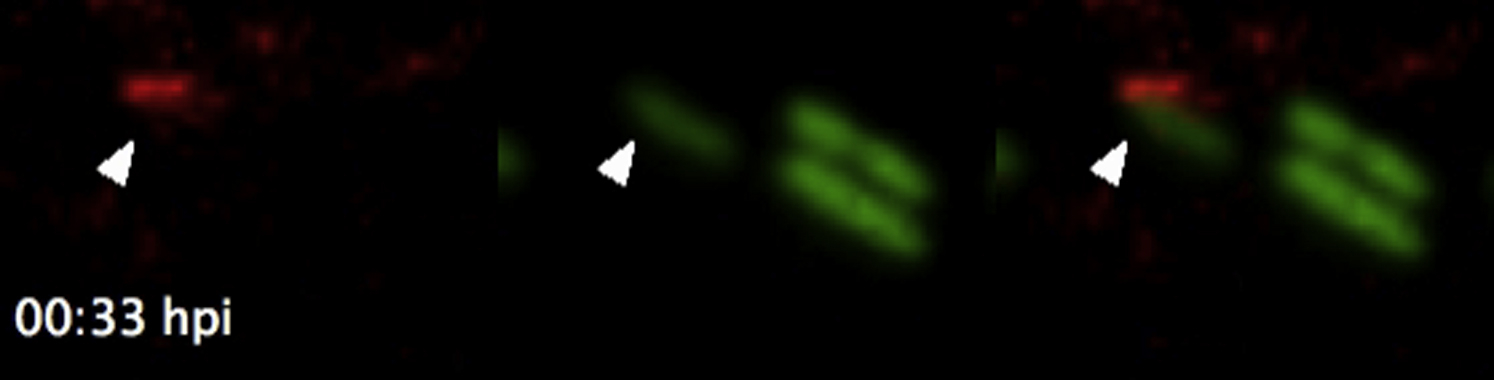

Supplement: Movie S2. Predation of Shigella by Bdellovibrio Inside a Zebrafish Larva, Related to Figure 2 — WT AB zebrafish larvae were injected in the tail muscle with 103 GFP-S. flexneri followed by a tail muscle injection of 1-2 × 10^5^ PFU mCherry-Bdellovibrio 30–90 min after initial Shigella infection. Single cell bacterial interactions were visualized at the infection site by confocal microscopy at 63× magnification. Invasion of Shigella (green) by Bdellovibrio (red) shown here. Z stacks were acquired at 3 min intervals. [file mmc3.jpg]

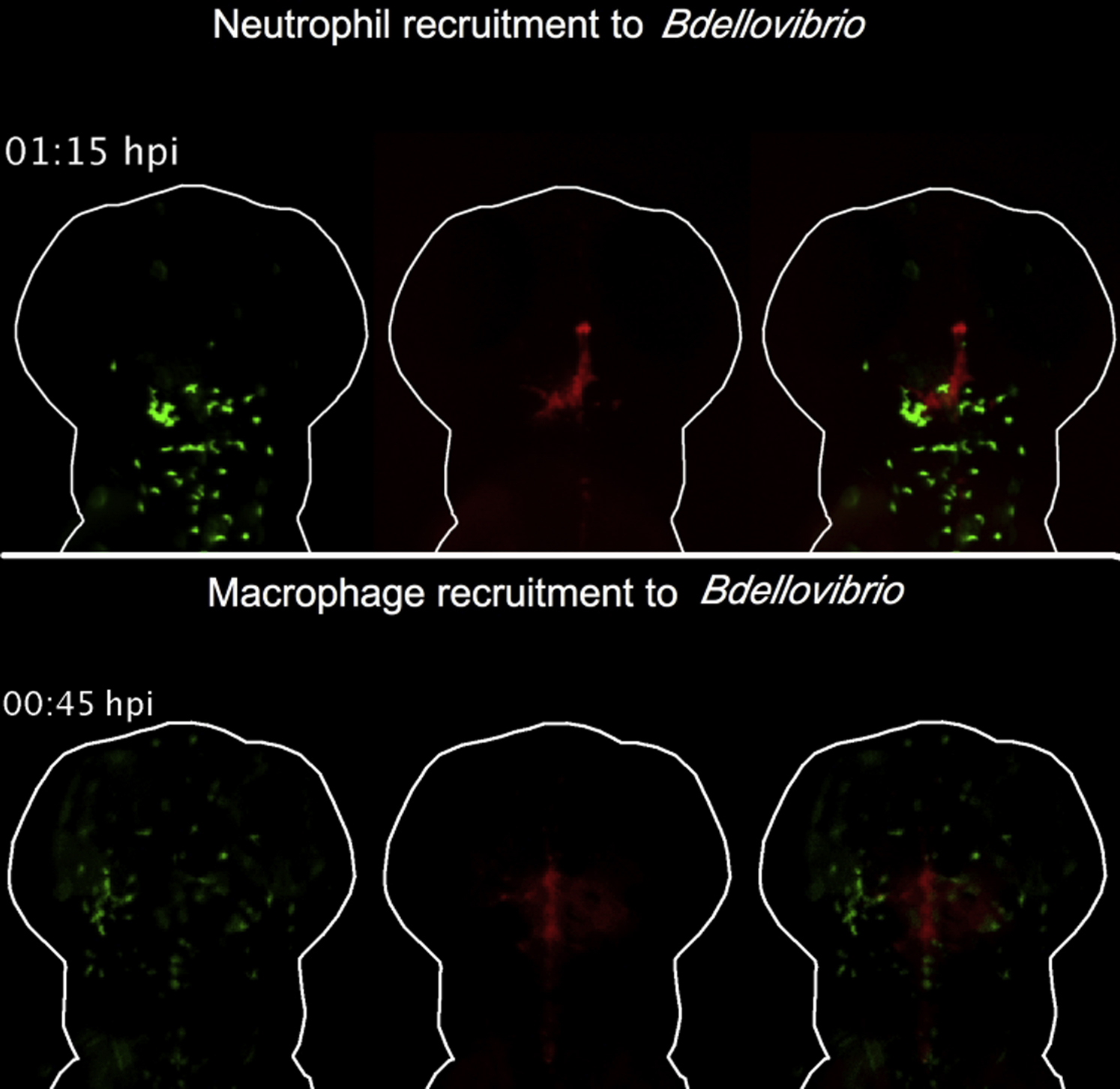

Supplement: Movie S3. Recruitment of Leukocytes to Bdellovibrio In Vivo, Related to Figure 3 — 1-2 × 10^5^ PFU mCherry-Bdellovibrio (for neutrophil interactions) or mTeal-Bdellovibrio (for macrophage interactions) were injected into the hindbrain ventricle of transgenic zebrafish larvae at 3 dpf. Tg(mpx:GFP)i114 larvae were used to visualise neutrophils. Tg(mpeg1:Gal4-FF)gl25;/Tg(UAS-E1b:nfsB.mCherry)c264 were used to visualise macrophages. Interactions between Bdellovibrio (red) and either neutrophils (green) or macrophages (green) were visualised by fluorescent stereomicroscopy. Z-stacks were acquired at 15 or 5 min intervals. [file mmc4.jpg]

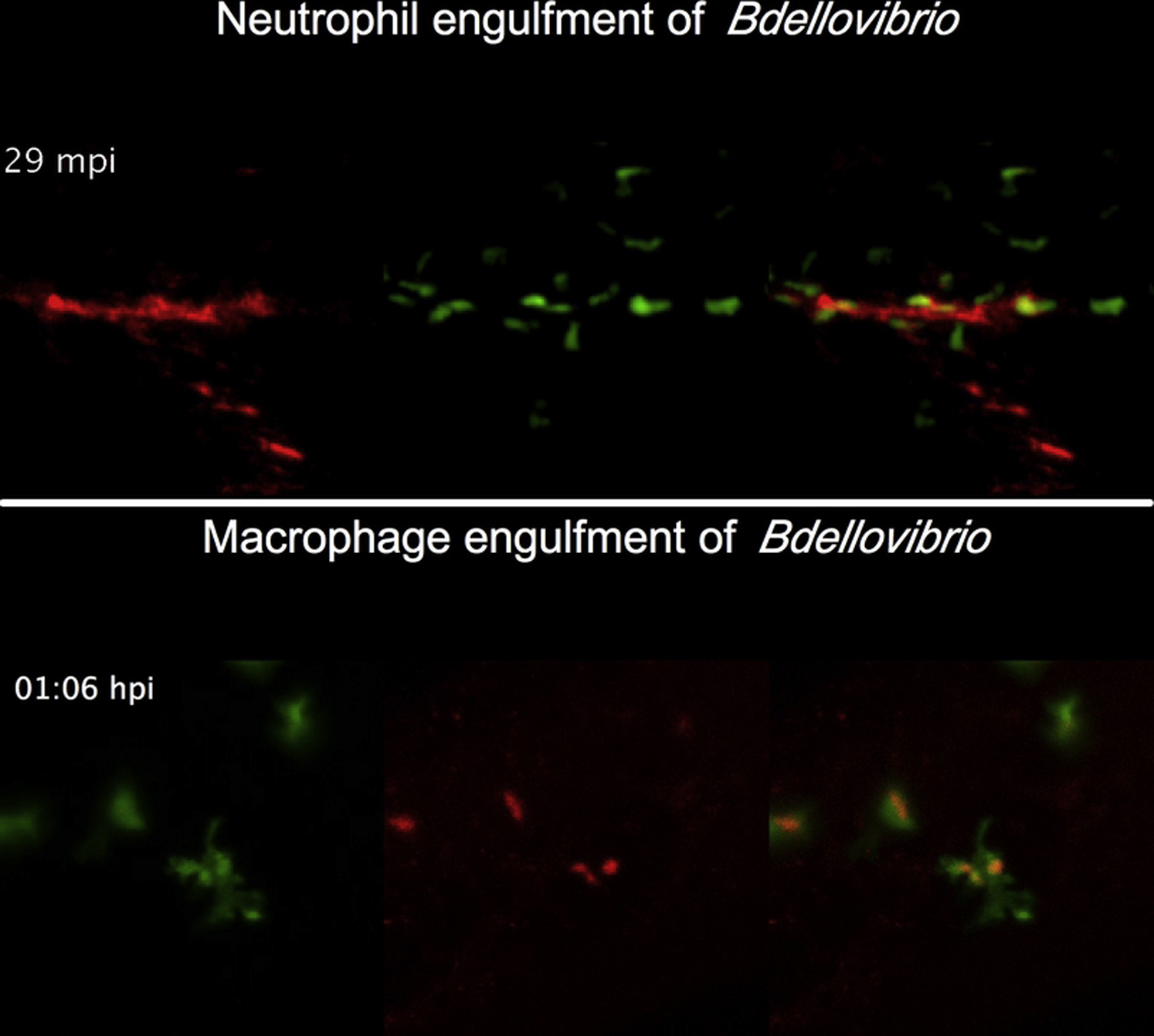

Supplement: Movie S4. Leukocyte Engulfment of Bdellovibrio In Vivo, Related to Figure 3 — 1-2 × 10^5^ PFU mCherry-Bdellovibrio (for neutrophil interactions) or mTeal-Bdellovibrio (for macrophage interactions) were injected into the tail muscle of transgenic zebrafish larvae at 3 dpf. Tg(mpx:GFP)i114 larvae were used to visualise neutrophils. Tg(mpeg1:Gal4-FF)gl25/Tg(UAS-E1b:nfsB.mCherry)c264 were used to visualize macrophages. Interactions between Bdellovibrio (red) and either neutrophils (green) or macrophages (green) were visualized by confocal microscopy at 40× magnification. Z stacks were acquired at 3 or 2 min intervals. [file mmc5.jpg]
