# Supplementary material for: Injections of Predatory Bacteria Work Alongside Host Immune Cells to Treat Shigella Infection in Zebrafish Larvae
Source: Curr Biol. 2016 Dec 19;26(24):3343–51. doi: 10.1016/j.cub.2016.09.067 (PMC5196024; doi:10.1016/j.cub.2016.09.067)
Supplement: Document S2. Article plus Supplemental Information [file mmc6.pdf]

Report

Current Biology

Injections of Predatory Bacteria Work Alongside Host Immune Cells to Treat *Shigella* Infection in Zebrafish Larvae

Highlights

- Injected predatory *Bdellovibrio* bacteria persist non-pathogenically in zebrafish
- *Bdellovibrio* injection promotes *Shigella* killing and increases zebrafish survival
- *Bdellovibrio* are eventually cleared by the zebrafish immune system
- Antibacterial therapy is achieved via the host immune system working with *Bdellovibrio*

Authors

Alexandra R. Willis, Christopher Moore, Maria Mazon-Moya, ..., Robert Till, Serge Mostowy, R. Elizabeth Sockett

Correspondence

s.mostowy@imperial.ac.uk (S.M.), liz.sockett@nottingham.ac.uk (R.E.S.)

In Brief

In an era of global antibiotic resistance, Willis et al. characterize the "living-antibiotic" action of predatory *Bdellovibrio* bacteria in zebrafish larvae versus the human pathogen *Shigella flexneri*. Results are proof of principle that predators assist the immune system to promote animal survival upon infection by Gram-negative pathogens.

CrossMark

Willis et al., 2016, Current Biology *26*, 3343–3351
December 19, 2016 © 2016 The Authors. Published by Elsevier Ltd.
http://dx.doi.org/10.1016/j.cub.2016.09.067

CellPress

Current Biology
Report

CellPress

Injections of Predatory Bacteria Work Alongside Host Immune Cells to Treat *Shigella* Infection in Zebrafish Larvae

Alexandra R. Willis,[1] Christopher Moore,[2] Maria Mazon-Moya,[1] Sina Krokowski,[1] Carey Lambert,[2] Robert Till,[2] Serge Mostowy,[1,3,*] and R. Elizabeth Sockett[2,*]
[1]MRC Centre of Molecular Bacteriology and Infection (CMBI), Imperial College London, London SW7 2AZ, UK
[2]School of Life Sciences, Nottingham University Medical School, QMC, Derby Road, Nottingham NG7 2UH, UK
[3]Lead Contact
*Correspondence: s.mostowy@imperial.ac.uk (S.M.), liz.sockett@nottingham.ac.uk (R.E.S.)
http://dx.doi.org/10.1016/j.cub.2016.09.067

SUMMARY

***Bdellovibrio bacteriovorus* are predatory bacteria that invade and kill a range of Gram-negative bacterial pathogens in natural environments and in vitro [1, 2]. In this study, we investigated *Bdellovibrio* as an injected, antibacterial treatment in vivo, using zebrafish (*Danio rerio*) larvae infected with an antibiotic-resistant strain of the human pathogen *Shigella flexneri*. When injected alone, *Bdellovibrio* can persist for more than 24 hr in vivo yet exert no pathogenic effects on zebrafish larvae. *Bdellovibrio* injection of zebrafish containing a lethal dose of *Shigella* promotes pathogen killing, leading to increased zebrafish survival. Live-cell imaging of infected zebrafish reveals that *Shigella* undergo rounding induced by the invasive predation from *Bdellovibrio* in vivo. Furthermore, *Shigella*-dependent replication of *Bdellovibrio* was captured inside the zebrafish larvae, indicating active predation in vivo. *Bdellovibrio* can be engulfed and ultimately eliminated by host neutrophils and macrophages, yet have a sufficient dwell time to prey on pathogens. Experiments in immune-compromised zebrafish reveal that maximal therapeutic benefits of *Bdellovibrio* result from the synergy of both bacterial predation and host immunity, but that in vivo predation contributes significantly to the survival outcome. Our results demonstrate that successful antibacterial therapy can be achieved via the host immune system working together with bacterial predation by *Bdellovibrio*. Such cooperation may be important to consider in the fight against antibiotic-resistant infections in vivo.**

RESULTS AND DISCUSSION

Injected Predatory *Bdellovibrio* Persist in Zebrafish Larvae without Ill Effects and Treat *Shigella* Infection In Vivo

The rise in antimicrobial-resistant (AMR) Gram-negative bacterial infections in hospital patients has prompted an urgent search for novel antibacterial agents [3]. One candidate group is the naturally predatory bacteria *Bdellovibrio bacteriovorus*, which invade and kill a wide range of Gram-negative bacterial pathogens [1]. Invasion is followed by pathogen rounding, after which the prey pathogen dies [2]. *Bdellovibrio* replicate within the dead pathogen, which persists as a stable "bdelloplast" structure until being lysed 3–4 hr after invasion [4]. Prey lysis releases mature *Bdellovibrio* progeny that can seek further bacterial victims (Figure 1A).

Given that *Bdellovibrio* are ubiquitous in nature, it is likely that these bacteria are already being harmlessly ingested in food or water. Indeed, low levels of *Bdellovibrio* 16S rRNA have been detected in gut samples from healthy children [5]. Although no equivalent studies have been conducted in adults, there is no known association of *Bdellovibrio* with disease. Previously, non-injected administrations of *Bdellovibrio* have been shown to reduce pathogen numbers by oral administration versus *Salmonella enteritidis* in the gut of chickens [6] and by external application against eye infection in cattle [7–9]. Emerging global antibiotic resistance calls for new injected therapies to target infected wounds and body compartments. The capability of injected *Bdellovibrio* to treat bacterial infections has not been tested, nor has the interaction of predatory bacteria with leukocytes, and successes here could dramatically expand the therapeutic scope of predatory bacteria against life-threatening infections. It is, therefore, important to consider the extent to which injected *Bdellovibrio* survive in the presence of a vertebrate immune system and whether leukocyte interactions aid or prevent pathogen killing in vivo. Measuring these parameters is crucial to develop the therapeutic potential of *Bdellovibrio*.

The transparent zebrafish larva provides a unique opportunity to quantify and visualize in vivo both the spread or restriction of bacterial infection as well as bacterial interactions with immune cells [10]. In particular, the zebrafish hindbrain ventricle is highly amenable to imaging, enabling us to follow injected *Bdellovibrio* ± pathogenic bacteria over time (Figure S1A). To first test for any pathogenic effects of *Bdellovibrio* inside a vertebrate host, we injected the hindbrain ventricle of zebrafish larvae at 3 days post-fertilization (dpf) with 1–10 × 10^4 plaque-forming units (PFUs) of mCherry *B. bacteriovorus* HD100 alone. This assessment was also essential to determine whether injected *Bdellovibrio* alone would survive in the zebrafish long enough to successfully prey on pathogen infections. Live-cell imaging showed a gradual reduction in fluorescence from

This is an open access article under the CC BY license (http://creativecommons.org/licenses/by/4.0/).

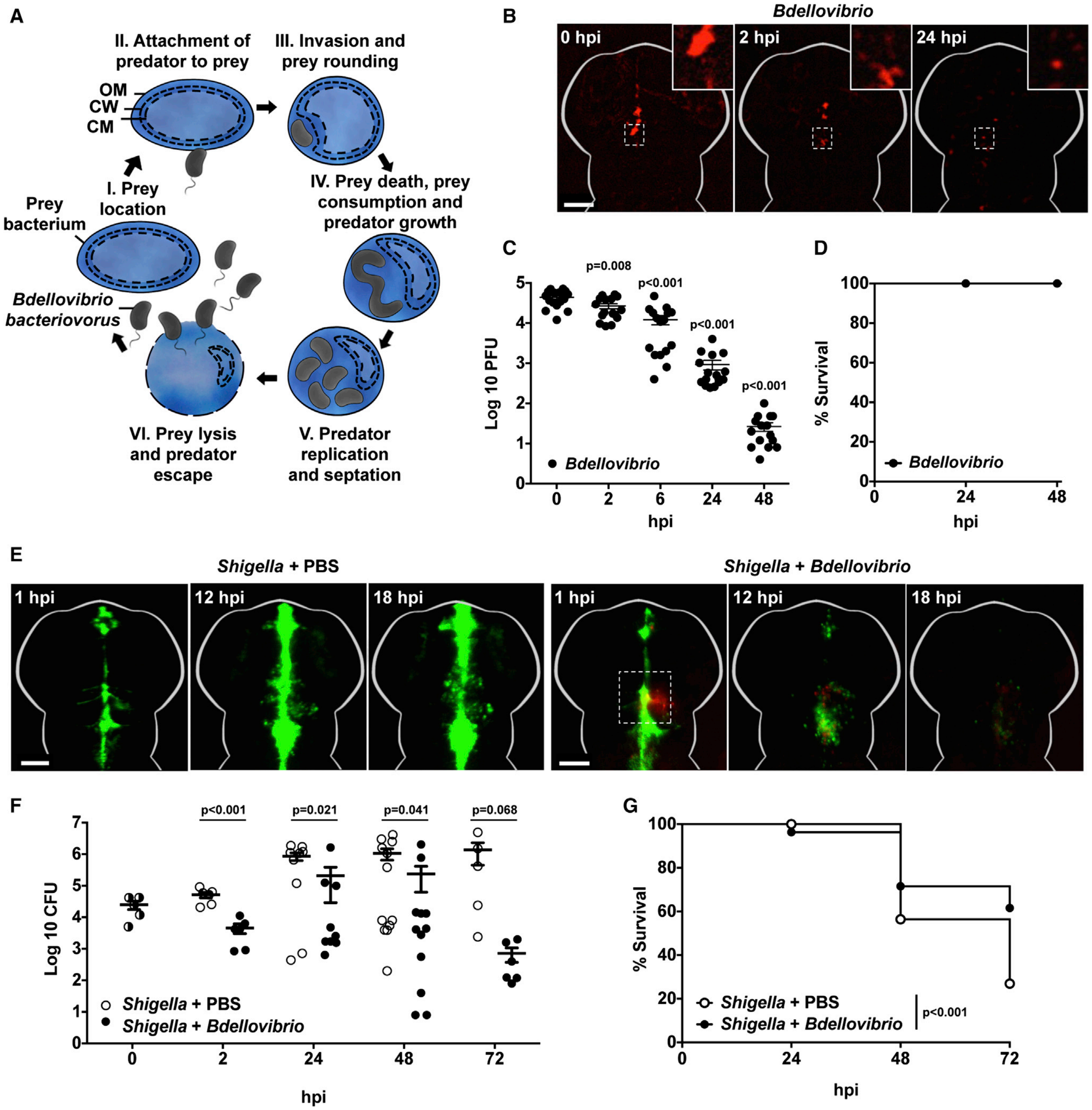

Figure 1. Injected Predatory *Bdellovibrio* Persist in Zebrafish Larvae without Ill Effects and Protect against *Shigella* Infection In Vivo

(A) Cartoon of *Bdellovibrio* life cycle. (I–III) Motile predatory *Bdellovibrio* attach to and invade the periplasm of Gram-negative bacteria such as *Shigella*. (III) Prey bacteria are rounded by DD-endopeptidase action on the cell wall. (IV) Prey bacteria are killed in ~30 min and kept intact as *Bdellovibrio* consume their contents and grow. (V and VI) Following replication, *Bdellovibrio* lyse prey 180–240 min after invasion, releasing further predators. These *Bdellovibrio* progeny can repeat the predatory cycle. OM, outer membrane; CW, cell wall peptidoglycan; CM, cytoplasmic membrane.

(B) Wild-type (WT) AB larvae were injected at 3 dpf in the hindbrain ventricle with 1–10 × 10^4 PFUs of mCherry-*Bdellovibrio* (red). The same larvae were imaged over time to observe distribution. Representative images from a single larva are shown here. Scale bar, 100 μm.

(C) Enumeration of live *Bdellovibrio* in PBS-homogenates from larvae injected with mCherry-*Bdellovibrio* as in (B) over time. Each circle represents a count from an individual larva. Data are pooled from two independent experiments (n = 8 larvae per experiment). Mean ± SEM (horizontal bars) is shown. The p values (versus the 0 hpi time point) were determined by multiple t test. Significance with Bonferroni correction was defined as $p < 0.0125$. See also Figures S1B–S1D for comparative evaluations of *Bdellovibrio* persistence from different doses in larvae at different developmental stages.

(D) Survival curve of WT AB larvae injected with mCherry-*Bdellovibrio* as in (B) and incubated at 28°C for 48 hpi. Data are pooled from three independent experiments (n = 22–37 larvae per experiment).

(legend continued on next page)

mCherry-*Bdellovibrio* following injection, although some bacteria were clearly observed in vivo after 24 hr post-infection (hpi) (Figure 1B). For quantification of the survival of *Bdellovibrio* in vivo, injected larvae were homogenized and predatory bacteria enumerated over time post-infection (Figure 1C). Consistent with observations from live-cell imaging, we detected a steady decline in *Bdellovibrio* numbers from 2 hpi, and by 48 hpi the bacteria were largely eliminated. Similar results were obtained using a 10-fold higher dose of *Bdellovibrio* or in larvae injected 2 dpf when the immune system is less developed (Figures S1B–S1D). These results reflect the obligatory prey-dependent lifestyle of *Bdellovibrio*. They are able to survive within zebrafish for extended periods of time but are unable to replicate in the absence of prey bacteria, giving an opportunity for *Bdellovibrio* to act therapeutically before ultimately being cleared. Moreover, *Bdellovibrio*-injected zebrafish larvae displayed normal morphology and locomotive activity, with no signs of developmental toxicity or reduced viability (Figures 1D and S1E). These results raise no concern for the use of *Bdellovibrio* as a therapeutic agent in vivo, and its natural clearance could be viewed as a beneficial property of a limited treatment.

Given that *Bdellovibrio* have no adverse effects on zebrafish, we tested their efficacy as an antibacterial agent against a streptomycin- and carbenicillin-resistant strain of *Shigella flexneri* M90T. *Shigella* is a Gram-negative enteroinvasive pathogen that is responsible for 163 million illness episodes and over 1 million deaths annually [11]. Similar to many other Gram-negative pathogens in hospital patients, cases of antibiotic-resistant *Shigella* are rising [12]. *Bdellovibrio* invade Gram-negative pathogens without a simple receptor-based recognition system, making *Bdellovibrio* resistance genetically complex for prey to acquire. Previous studies have used the zebrafish as a model organism to study the infection biology of *S. flexneri* M90T infection in vivo [13, 14]. We developed this model of *Shigella* infection to incorporate the additional injection of *B. bacteriovorus* and test its therapeutic potential. Here, 30–90 min after an otherwise lethal (at 48–72 hpi) hindbrain inoculation of GFP-*Shigella* M90T (>5×10^3 colony-forming units [CFUs]), we injected $1–2 \times 10^5$ PFUs of mCherry-*Bdellovibrio* into the hindbrain ventricle of zebrafish larvae. In the absence of *Bdellovibrio* injection, zebrafish larvae showed increasing fluorescence of GFP-*Shigella* (Figure 1E; Movie S1). Strikingly, larvae injected with *Bdellovibrio* showed diminishing fluorescence of GFP-*Shigella* in regions contacting mCherry-*Bdellovibrio* (Figure 1E; Movie S1). Consistent with this, *Shigella* enumeration demonstrated that zebrafish larvae injected with *Bdellovibrio* were able to control *Shigella* replication significantly better than those infected with *Shigella* alone (Figure 1F). Moreover, *Bdellovibrio* could rescue zebrafish from lethal *Shigella* infection, increasing survival by ~35% at 72 hpi (Figure 1G).

Bdellovibrio Prey on *Shigella* In Vitro and In Vivo inside Living Zebrafish

To confirm susceptibility of *Shigella* to *Bdellovibrio* predation, we performed co-incubation assays in vitro, and we observed mCherry-*Bdellovibrio* invade GFP-*Shigella* and, subsequently, induce prey rounding to form bdelloplasts (Figure 2A). In live-cell counting assays of viability in buffer, *Shigella* numbers were reduced >4,000-fold by *Bdellovibrio* treatment (Figure 2B). Plate reader assays measuring optical density of *Shigella* and fluorescence of mCherry-*Bdellovibrio* confirmed that reduction in *Shigella* numbers was followed by a rise in *Bdellovibrio* numbers after an ~3 hr predatory cycle, during which they invade and grow within *Shigella* and then emerge (Figures 2C and 2D).

To investigate *Bdellovibrio* predation of *Shigella* in vivo, we imaged the zebrafish tail muscle after sequential GFP-*Shigella* and mCherry-*Bdellovibrio* co-injections. *Shigella* are rod-shaped bacteria (~0.5 × 2.0 μm); however, *Shigella* in the presence of *Bdellovibrio* were mostly rounded (~1.0 × 1.0 μm), suggesting their invasion by *Bdellovibrio* inside zebrafish (Figure 2E). To investigate this further, we followed in vivo predator-prey interactions at the level of single cells (Figure 2F). Remarkably, confocal microscopy inside live zebrafish confirmed that mCherry-*Bdellovibrio* invade individual GFP-*Shigella* to form green rounded bdelloplasts containing red *Bdellovibrio* over a similar time frame to that seen in vitro (Movie S2). To test for *Bdellovibrio* predation of *Shigella* at the whole-animal level, we quantified *Bdellovibrio* over time after sequential GFP-*Shigella* and mCherry-*Bdellovibrio* co-injections (Figure 2G). As expected from experiments performed both in vitro (Figure 2D) and in vivo (Figure 1C), in the absence of *Shigella*, prey zebrafish larvae showed decreasing numbers of *Bdellovibrio* over the 24 hr following infection (Figure 2G). In contrast, zebrafish larvae infected with *Shigella* and *Bdellovibrio* showed significantly increased numbers of *Bdellovibrio* at 5 hpi, indicating predatory replication inside *Shigella* in vivo. Thus, live bacterial predation is occurring within zebrafish. To dissect the efficacy of *Bdellovibrio* therapy in the context of innate immunity, we next tested how leukocytes can affect the efficacy of *Bdellovibrio* predation in our zebrafish infection model.

(E) WT AB zebrafish larvae were injected in the hindbrain ventricle at 3 dpf with >5×10^3 CFUs of GFP-*S. flexneri* (green), followed by a hindbrain injection of either PBS or $1–2 \times 10^5$ PFUs of mCherry-*Bdellovibrio* (red), 30–90 min after the initial *Shigella* infection. Representative images of the hindbrain ventricle in PBS- or *Bdellovibrio*-treated zebrafish larvae infected with *Shigella* are shown. Dotted square shows region of interaction between fluorescent *Shigella* and *Bdellovibrio*. For each treatment, the same larva was imaged over time. Scale bar, 100 μm. See also Movie S1.

(F) Enumeration of live *Shigella* in homogenates of larvae injected with *S. flexneri* and treated with injections of either PBS or *Bdellovibrio* as in (E) over time. Each circle represents a count from an individual larva. Half-filled circles represent enumerations from larvae at time 0 and are representative of inocula for both conditions. Only viable larvae were included in the analysis. Data are pooled from four independent experiments (up to n = 3 larvae per time point per experiment). Mean ± SEM (horizontal bars) is shown. The p values (between conditions at cognate time points) were determined by unpaired one-tailed Student's t test. Significance was defined as $p < 0.05$.

(G) Survival curve of larvae injected with *S. flexneri* and treated with either PBS or *Bdellovibrio* as in (E). Larvae were incubated at 28°C for 72 hpi. Data are pooled from three independent experiments (n = 22–48 larvae per condition per experiment). Up to three larvae per condition were taken for CFUs at 2, 24, 48, and 72 hr time points. The p value between conditions was determined by log-rank Mantel-Cox test. Significance was defined as $p < 0.05$.

See also Figure S1.

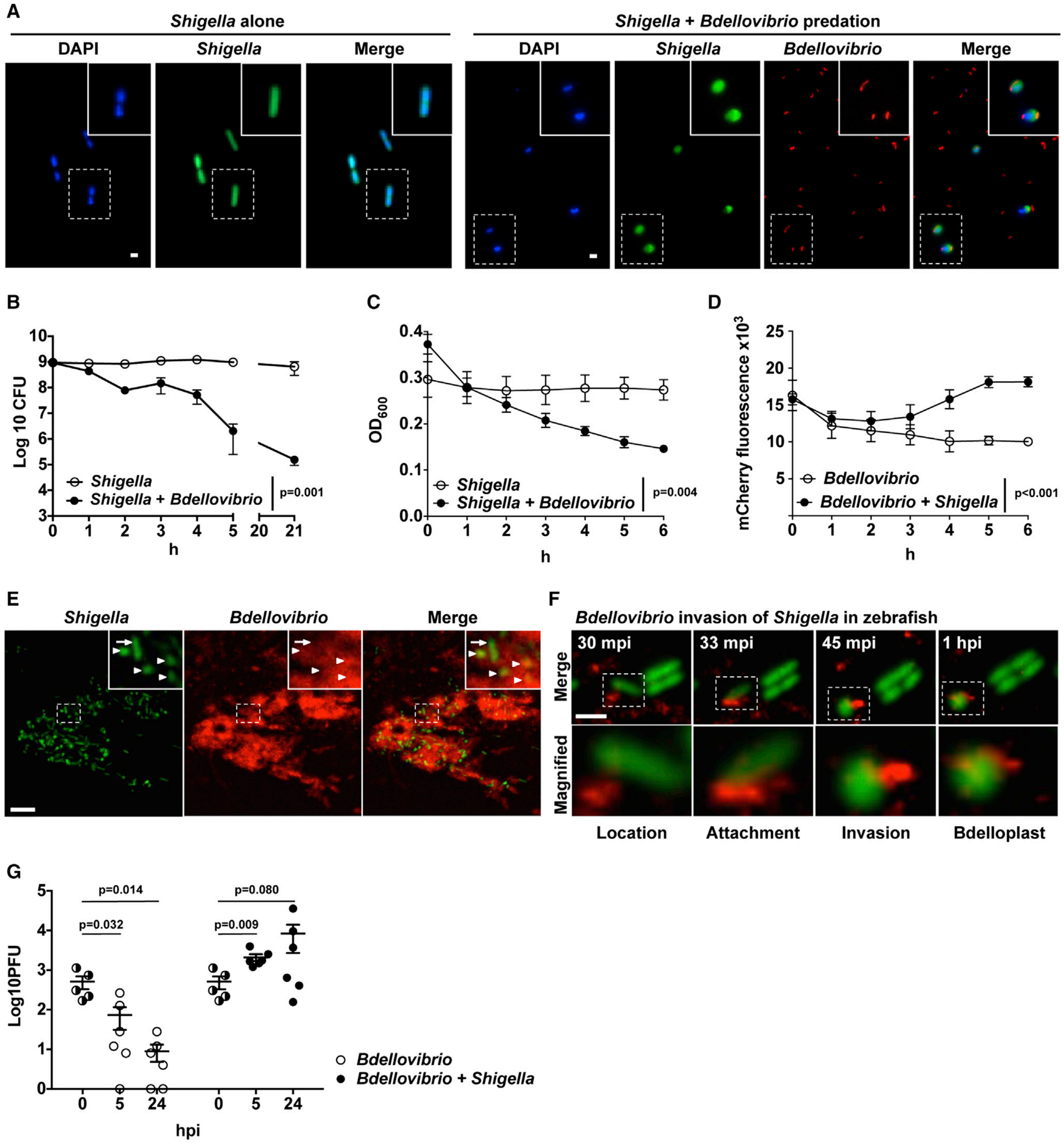

Figure 2. *Bdellovibrio* Prey on *Shigella* In Vitro and In Vivo inside Living Zebrafish

(A) GFP-*S. flexneri* (green) were incubated in vitro, in the presence or absence of mCherry-*Bdellovibrio* (red), and visualized by wide-field fluorescent microscopy. Representative images, including rod-shaped *Shigella* and rounded *Shigella* invaded by smaller comma-shaped *Bdellovibrio*, were taken at 1 hr post-mixing. Scale bar, 1 μm.

(B) 5–12 × 10^8 CFUs of GFP-*S. flexneri* were incubated, in vitro, in 10 mL CaHEPES buffer for 21 hr in the presence or absence of ~6.2 × 10^{10} PFUs of mCherry-*Bdellovibrio*. Live *Shigella* were enumerated over time. Data are pooled from three independent experiments. Mean ± SEM (horizontal bars) is shown. The p value between conditions was determined by paired one-tailed Student's t test. Significance was defined as $p < 0.05$.

(C and D) 2–7 × 10^7 CFUs of GFP-*S. flexneri*, 8.4–10.4 × 10^9 PFUs of mCherry-*Bdellovibrio*, or both GFP-*S. flexneri* and mCherry-*Bdellovibrio* were incubated in vitro in CaHEPES buffer at 37°C. (C) Optical density 600 (OD_{600}) representing *Shigella* numbers (*Bdellovibrio* are too small to contribute to OD_{600}) or (D) mCherry fluorescence intensity representing *Bdellovibrio* numbers was measured every 30 min for 6 hr using a microplate reader (results plotted every 1 hr). Mean ± SEM

(legend continued on next page)

Bdellovibrio Is Recognized and Engulfed by Zebrafish Leukocytes In Vivo

Time-lapse microscopy in the hindbrain showed that, by 6 hr following injection, the initially dispersed mass of mCherry-*Bdellovibrio* clustered and formed dynamic punctae of $\sim$10 μm. Similar observations were made after caudal vein injection of *Bdellovibrio* into the bloodstream of zebrafish larvae (Figure S2A). These observations suggest that *Bdellovibrio* can reside within leukocytes. Although little studied, some features of *Bdellovibrio* have been predicted to allow a degree of "silent running" in the immune system of a vertebrate. *Bdellovibrio* are characteristically small, 0.25 × 1.0 μm, comprise a single-membrane-sheathed flagellum, and have a modified mannosylated lipopolysaccharide (LPS) outer membrane [15]. Furthermore, *Bdellovibrio* gene expression and surface protein production are significantly lower outside of bacterial prey hosts than inside [16, 17]. To test for innate immune detection of *Bdellovibrio* in vivo, we injected larvae with mCherry-*Bdellovibrio*, and quantified the expression of the pro-inflammatory cytokines interleukin 1 β (*il1b*) and tumor necrosis factor α (*tnf*-α) by qRT-PCR. Increased expression of both *il1b* and *tnf*-α was detected 4 hr after larval inoculation of *Shigella*, *Bdellovibrio*, or *Shigella* + *Bdellovibrio* combined (Figure S2B). Importantly, the cytokine response from *Shigella* + *Bdellovibrio* together is not additive beyond *Shigella* alone, demonstrating that *Bdellovibrio* is not solely stimulating a further immune response to help clear pathogenic bacteria.

Cytokine signaling during zebrafish infection is typically accompanied by an active immune cell response [18, 19]. The innate immune system of zebrafish is highly homologous to that of humans, and responses are mediated by neutrophils and macrophages [19]. Leukocytes do not typically reside in the hindbrain during steady-state conditions, making this site ideal to study directed leukocyte migration in response to injected bacteria. To test *Bdellovibrio* interactions with leukocytes, we used Tg(*mpx*:GFP)^{i114} transgenic larvae with GFP-neutrophils and Tg(*mpeg1*:Gal4-FF)^{gl25}/Tg(UAS-E1b:nfsB.mCherry)^{c26} transgenic larvae with mCherry-macrophages. Consistent with a stimulated cytokine response (Figure S2B), imaging of *Bdellovibrio* hindbrain injections in zebrafish with these fluorescent leukocytes revealed that both neutrophils and macrophages localize to the site of injection (Figures 3A and 3B; Movie S3). Despite detection of *Bdellovibrio* by innate immune cells in vivo, quantification of leukocyte recruitment to the larval head, via image analysis, revealed only a slight increase over PBS controls in neutrophils (1.5-fold ± 0.1) and macrophages (1.2-fold ± 0.1) in the hindbrain at 6 hpi (Figures 3C and 3D).

Analysis by confocal microscopy of *Bdellovibrio*-leukocyte interactions within live zebrafish confirmed that these bacteria are engulfed by both neutrophils and macrophages (Figures S2C and S2D; Movie S4). To assess the role of leukocytes in *Bdellovibrio* clearance, we performed experiments in immunocompromised zebrafish larvae using an antisense morpholino oligonucleotide targeting Pu.1, a zebrafish transcription factor driving myeloid gene expression [20]. Larvae depleted of leukocytes, via prior injection of Pu.1-targeting morpholino into the one- to eight-cell-stage embryo, were injected at 3 dpf in the hindbrain with 1–2 × 10^5 PFUs of *Bdellovibrio*. In agreement with a role for leukocytes in the clearance of *Bdellovibrio* in vivo, significantly more predatory bacteria were recovered from larval homogenates of Pu.1 morphants as compared to controls (Figures 3E and S2E). Survival of control or Pu.1 morphants injected with *Bdellovibrio* was not significantly different from each other (Figure S2F), highlighting that prolonged exposure to *Bdellovibrio* is not detrimental to the health of an immune-compromised animal.

Bdellovibrio Work alongside Innate Immune Cells to Protect Against *Shigella* Infection In Vivo

To understand better the relative contributions of *Bdellovibrio* and the host immune system to *Shigella* clearance, we performed infection studies in zebrafish larvae in which leukocytes were depleted by Pu.1 morpholino. Control and Pu.1 morphants were injected with lethal hindbrain doses of *Shigella* as before and treated with PBS or *Bdellovibrio* (Figure S3A). Enumerations of *Shigella* from larval homogenates showed that treatment with *Bdellovibrio* reduced *Shigella* numbers in both immune-compromised and immune-competent zebrafish larvae (Figure 4A), but survival was significantly greater in immune-competent larvae (Figure 4B). Remarkably, these results show that maximal therapeutic benefit of *Bdellovibrio* against a Gram-negative bacterial infection is ultimately the product of eukaryotic leukocytes working cooperatively with prokaryotic predators.

from three biological replicates with three technical replicates each is shown. The p value between conditions was determined by paired one-tailed Student's t test. Significance was defined as $p < 0.05$.

(E) WT AB zebrafish larvae were injected at 3 dpf in the tail muscle with 10^3 CFUs of GFP-*S. flexneri* (green) followed by a tail muscle injection of 1–2 × 10^5 PFUs of mCherry-*Bdellovibrio* (red) 30–90 min after the initial *Shigella* infection. Larvae were imaged by confocal microscopy at 20× magnification. Representative images show the different morphologies of *Shigella* in vivo, including the typical rod-shaped *Shigella* (arrow) and also a high proportion of rounded *Shigella* (arrowheads) at regions of interaction with *Bdellovibrio*. Scale bar, 10 μm.

(F) Representative images of predation of *Shigella* by *Bdellovibrio* in vivo, inside a larva injected as in (E) and imaged by high-resolution confocal microscopy at 63× magnification. Frames captured over time show stages of *Bdellovibrio* (red) invasive predation and rounding of *Shigella* (green) in vivo. Scale bar, 2.5 μm. mpi, minutes post-infection. See also Movie S2.

(G) WT AB zebrafish larvae were injected in the hindbrain ventricle at 3 dpf with 2–6 × 10^5 CFUs of GFP-*S. flexneri* (green) alone or followed by a hindbrain injection of 1–30 × 10^2 PFUs of mCherry-*Bdellovibrio* (red) 30–90 min after the initial *Shigella* infection. *Bdellovibrio* were diluted 100-fold from usual injections to facilitate enumeration of any replicated predators. Enumeration of live *Bdellovibrio* in PBS-treated homogenates of larvae over time is shown. Each circle represents a count from an individual larva. Half-filled circles represent enumerations from larvae at time 0 and are representative of inocula for both conditions. Only viable larvae were included in the analysis. Data are pooled from two independent experiments (up to n = 3 larvae per time point per experiment). Mean ± SEM (horizontal bars) is shown. The p values (versus the 0 hpi time point) were determined by multiple t test. Significance with Bonferroni correction was defined as $p < 0.0125$. Of note, p values (not displayed on figure) between conditions at cognate time points were determined by unpaired one-tailed Student's t test with significance defined as $p < 0.05$. These are as follows: $p < 0.001$ between conditions at 5 hr and $p < 0.0852$ at 24 hr.

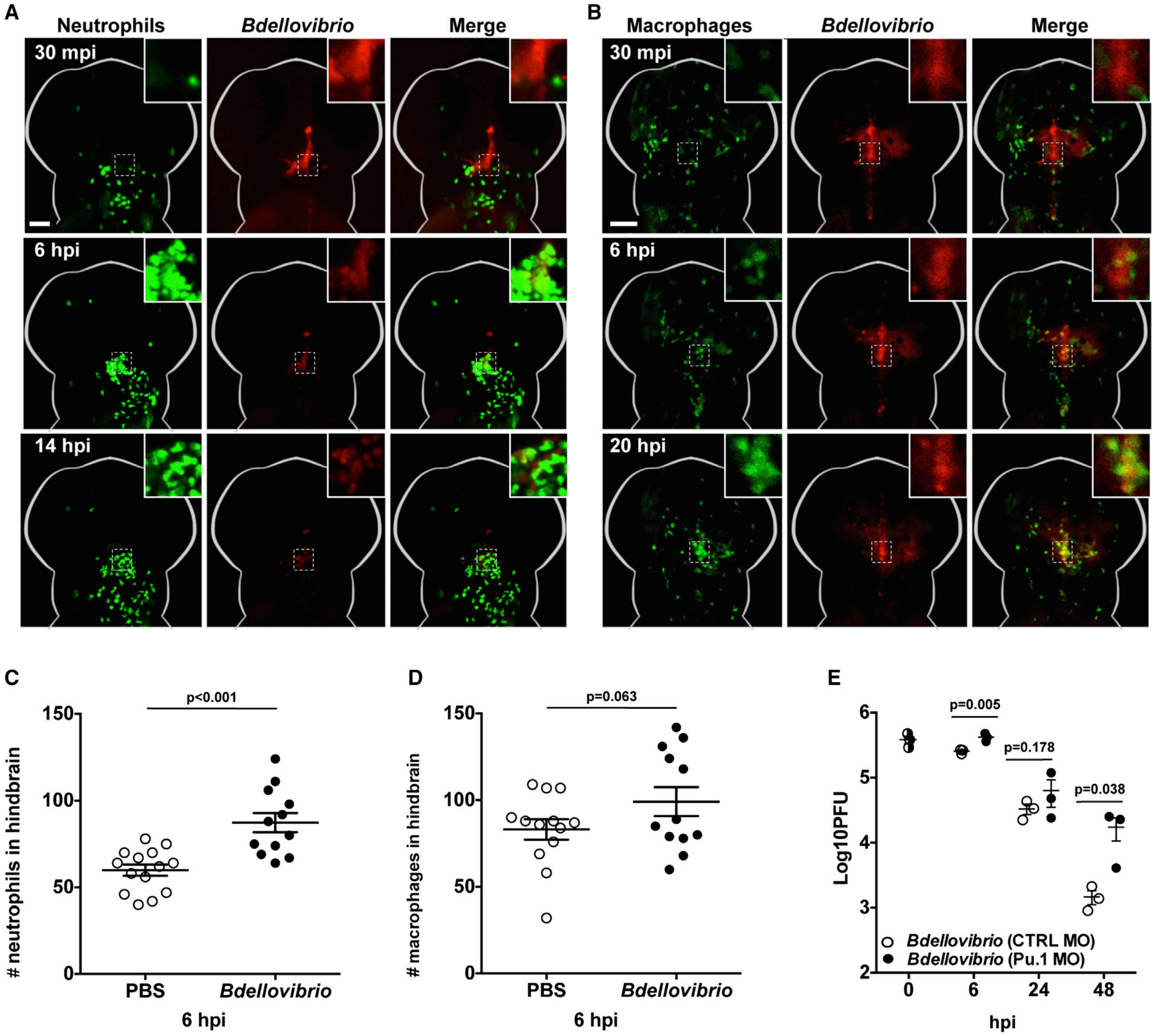

Figure 3. *Bdellovibrio* Is Recognized and Engulfed by Zebrafish Leukocytes In Vivo

(A) 1–2 × 10^5 PFUs of mCherry-*Bdellovibrio* were injected into the hindbrain ventricle of Tg(*mpx*:GFP)^{i114} zebrafish larvae at 3 dpf, and interactions between neutrophils (green) and *Bdellovibrio* (red) were visualized by fluorescent stereomicroscopy. Representative images from a single larva over time are shown. Scale bar, 100 μm. See also Movie S3.

(B) 1–2 × 10^5 PFUs of mTeal-*Bdellovibrio* were injected into the hindbrain ventricle of Tg(*mpeg1*:Gal4-FF)^{gl25}/Tg(UAS-E1b:nfsB.mCherry)^{c264} zebrafish larvae at 3 dpf, and interactions between macrophages (green) and *Bdellovibrio* (red) were visualized by fluorescent stereomicroscopy. Representative images from a single larva over time are shown. Scale bar, 100 μm. See also Movie S3.

(C) Tg(*mpx*:GFP)^{i114} zebrafish larvae were injected with PBS or *Bdellovibrio* as in (A), and GFP-expressing neutrophils present in the head region were quantified at 6 hpi. Each circle represents a count from an individual larva. Data are pooled from two independent experiments. The p value between conditions was determined by unpaired one-tailed Student's t test. Significance was defined as $p < 0.05$.

(D) Tg(*mpeg1*:Gal4-FF)^{gl25}/Tg(UAS-E1b:nfsB.mCherry)^{c264} zebrafish larvae were injected with PBS or *Bdellovibrio* as in (B), and mCherry-expressing macrophages present in the head region were quantified at 6 hpi. Each circle represents a count from an individual larva. Data are pooled from two independent experiments. The p value between conditions was determined by unpaired one-tailed Student's t test. Significance was defined as $p < 0.05$.

(E) Tg(*mpx*:GFP)^{i114} zebrafish larvae were pre-treated using control (CTRL) or Pu.1-targeting morpholino (MO) to deplete leukocytes. Morphants were injected in the hindbrain ventricle at 3 dpf with either PBS or 3–5 × 10^5 PFU mCherry-*Bdellovibrio*. Live *Bdellovibrio* were enumerated from PBS homogenates of larvae. Each circle represents a count from an individual larva. Half-filled circles represent enumerations from larvae at time 0 and are representative of inocula for both conditions. Mean ± SEM (horizontal bars) is shown. The p value (between conditions at cognate time points) was determined by unpaired one-tailed Student's t test. Significance was defined as $p < 0.05$. As inoculums from independent experiments were variable up to a log-fold, a representative of three independent experiments performed is shown. See also Figure S2E.

See also Figure S2.

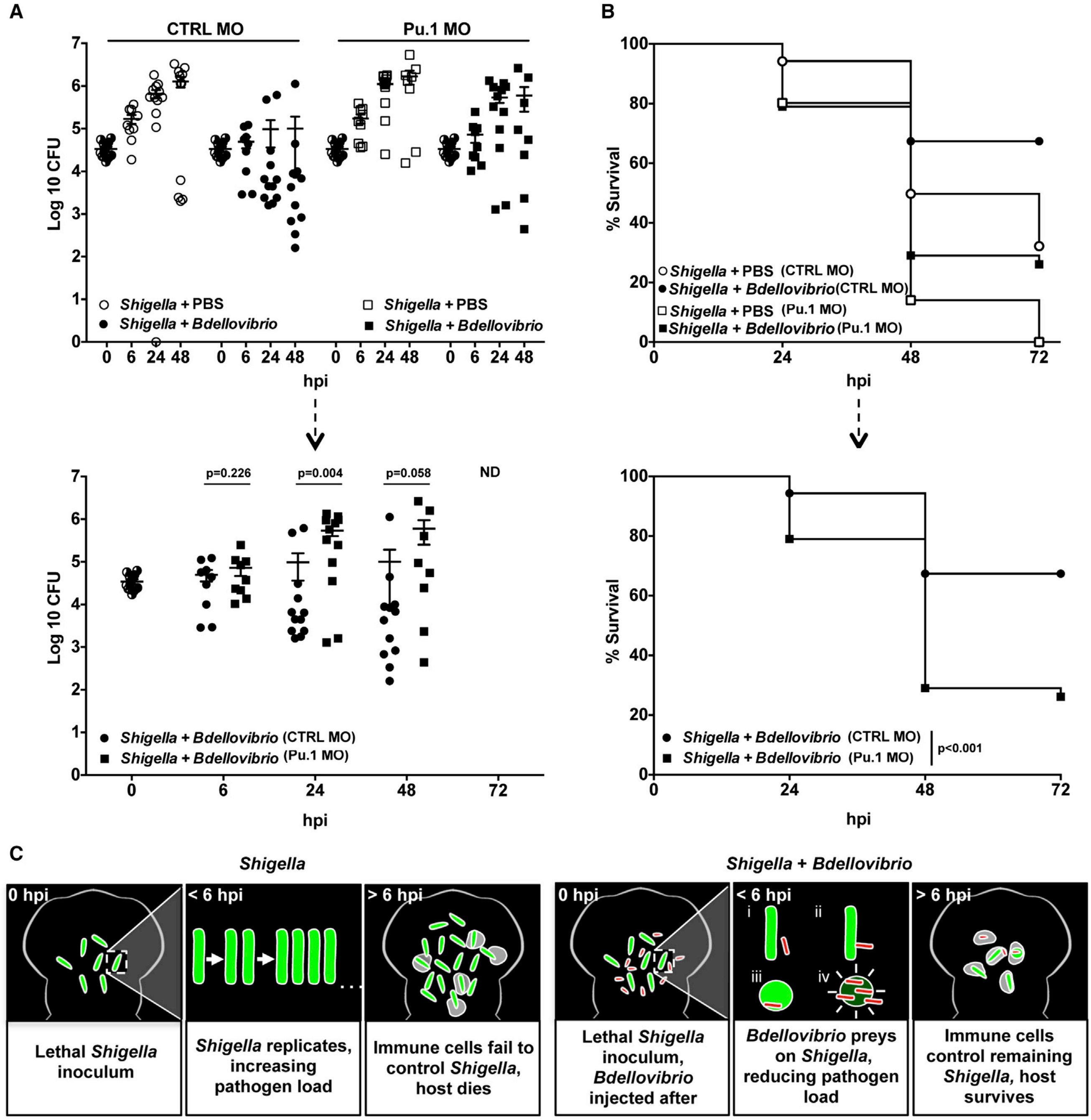

Figure 4. *Bdellovibrio* Work alongside Innate Immune Cells to Protect against *Shigella* Infection In Vivo

(A) Tg(*mpx*:GFP)^{i114} zebrafish larvae were pre-treated using control (CTRL) or Pu.1-targeting morpholinos (MO) to deplete leukocytes. Morphants were injected in the hindbrain ventricle at 3 dpf with >5 × 10^3 CFUs of GFP-*S. flexneri* followed by a hindbrain injection of PBS or 1–2 × 10^5 PFUs of mCherry-*Bdellovibrio* 30–90 min after the initial *Shigella* infection. Live *Shigella* were enumerated from larval homogenates. Each circle represents a count from an individual larva. Half-filled circles represent enumerations from larvae at time 0 and are representative of inocula for both conditions. Only viable larvae were included in the analysis. Data are pooled from three independent experiments (up to n = 3 larvae per time point per experiment). Mean ± SEM (horizontal bars) is shown. Top graph represents collated data. Bottom graph represents only *Bdellovibrio*-treated larvae, a subset of the above data. The p value (between conditions at cognate time points) was determined by unpaired one-tailed Student's t test. Significance was defined as $p < 0.05$. ND, not determined at 72 hpi due to high morphant mortality reducing the samples available.

(B) Survival curve of control (CTRL) or Pu.1 morphant larvae, injected with *S. flexneri* and treated with *Bdellovibrio* as in (A). Larvae were incubated at 28°C for 72 hpi. Data are pooled from three independent experiments (n = 12–40 larvae per condition per experiment). Up to three larvae per condition were taken for CFU at 6, 24, and 48 hr time points. Top graph represents collated data. Bottom graph represents only *Bdellovibrio*-treated larvae, a subset of the above data. The p value between conditions was determined by log-rank Mantel-Cox test. Significance was defined as $p < 0.05$.

(legend continued on next page)

The *Bdellovibrio* predatory process, which we have shown to be effective in vivo, could possibly allow treatment of infection sites in humans [21]. We propose that, following *Bdellovibrio* application, an early predatory-killing phase can reduce pathogen burden to a level manageable for clearance by the vertebrate innate immune system (Figure 4C). Predation will release small pathogen-derived fragments into the vertebrate, albeit in a digested state, after predator enzyme action. The limited immune stimulation by the injected *Bdellovibrio* (and possibly these pathogen fragments) is not detrimental to the well-being of zebrafish larvae. This limited immune stimulation may contribute to the clearance of *Shigella*, but active predation with predator-prey encounters in vivo occurs as early as 30 min post-injection (Figure 2F; Movie S1), time points before extensive leukocyte infiltration. Moreover, *Bdellovibrio* have replicated extensively in vivo at 5 hr, in a process dependent on *Shigella* killing (Figure 2G).

Predator-prey interactions in our experiments occurred early, when a significant percentage of pathogens would be outside of leukocytes. *Bdellovibrio* engulfed by leukocytes inside zebrafish were detectable by their mCherry fluorescence within the vacuoles of those leukocytes for up to 6 hr (Movie S4). This suggests that predatory bacteria may persist intracellularly within immune cells. Future studies beyond this work will be testing whether intracellular *Bdellovibrio* are able to access, invade, and kill Gram-negative pathogens, which themselves invade leukocytes, such as *Shigella* [22].

In conclusion, these results highlight the first successful use of *Bdellovibrio* in vivo as an injected antibacterial therapy, improving survival in live infected animals. The zebrafish infection model reveals host recognition and clearance of *Bdellovibrio* within days following treatment, a feature that provides a useful limitation to an applied therapy. Most importantly, we show that injected *Bdellovibrio* persist in vivo sufficiently long enough with predatory capacity to reduce numbers of pathogenic bacteria, before themselves being removed by immune action of the host.

In our study, the prokaryotic predator *Bdellovibrio* works together with the host immune system, which would otherwise be overwhelmed by a Gram-negative infection. These biological experiments suggest that when tackling pathogenic AMR bacterial infections in a human medical setting, active predation and any associated/limited immune-stimulatory side effects can be beneficial as long as patient physiology and well-being can be supported. Future experiments will allow us to characterize the host immune response in more detail, determine how predators can be prepared with modified immune-stimulatory properties, and examine how multiple doses of predators can be applied in more long-lived infections. The data in this study represent key milestones in future use of *Bdellovibrio* as a "living antibiotic" in vivo, and they warrant further research into the development of predatory bacteria as an antibacterial agent for infected sites or wounds in higher vertebrates and, ultimately, humans. The strength of such prokaryotic-predator: eukaryotic-leukocyte combinations is an important therapeutic consideration as we move forward in responding to new Gram-negative bacterial threats.

SUPPLEMENTAL INFORMATION

Supplemental Information includes Supplemental Experimental Procedures, three figures, and four movies and can be found with this article online at http://dx.doi.org/10.1016/j.cub.2016.09.067.

AUTHOR CONTRIBUTIONS

S.M. and R.E.S. acted jointly as senior authors of this collaboration; in line with journal policy, S.M. has been designated as lead contact as the majority of the work by all authors was performed at his lab. A.R.W., S.M., and R.E.S. designed the research and carried out analysis along with C.M. A.R.W., R.E.S., M.M.-M., C.M., C.L., and S.K. performed experiments with supervision and assistance from S.M. R.T. constructed the fluorescent *Bdellovibrio* strains, and A.R.W., C.M., M.M.-M., and S.M. carried out zebrafish husbandry. A.R.W., S.M., and R.E.S. wrote the manuscript with assistance from C.M. and input from all authors.

ACKNOWLEDGMENTS

S.M. and R.E.S. thank their laboratory members for the provision of cultures and reagents to support these experiments. We thank Michael Way, Clive Hoggart, and Vincenzo Torraca for helpful discussions as well as Antonio Pagan and Philip Elks for zebrafish tools. A.R.W. is supported by a Medical Research Council PhD studentship. Work in the S.M. laboratory is supported by a Wellcome Trust Research Career Development Fellowship (WT097411MA) and the Lister Institute of Preventive Medicine. Preliminary parts of this work were funded initially by Leverhulme Trust Research Leave Fellowship RF-2013-348 to R.E.S. Subsequent research including *Bdellovibrio* survival in larvae was sponsored by the US Army Research Office and the Defense Advanced Research Projects Agency (DARPA) under Cooperative Agreement W911NF-15-2-0028 to R.E.S. The views and conclusions contained in this document are those of the authors and should not be interpreted as representing the official policies, either expressed or implied, of the Army Research Office, DARPA, or the US government. Animal experiments conducted at Imperial College London and the University of Nottingham were performed according to the Animals (Scientific Procedures) Act 1986, and were approved by the Home Office under Project Licenses: PPL 70/7446 and PPL 30/3378, respectively.

Received: June 2, 2016
Revised: September 1, 2016
Accepted: September 30, 2016
Published: November 23, 2016

REFERENCES

1. Stolp, H., and Starr, M.P. (1963). *Bdellovibrio bacteriovorus* Gen. Et Sp. N., a predatory, ectoparasitic, and bacteriolytic microorganism. Antonie van Leeuwenhoek *29*, 217–248.
2. Sockett, R.E. (2009). Predatory lifestyle of *Bdellovibrio bacteriovorus*. Annu. Rev. Microbiol. *63*, 523–539.
3. Spellberg, B., Bartlett, J.G., and Gilbert, D.N. (2013). The future of antibiotics and resistance. N. Engl. J. Med. *368*, 299–302.

(C) Model for the therapeutic benefit of *Bdellovibrio* as an antibacterial agent against *S. flexneri* in vivo. The zebrafish immune system alone is unable to control high doses of *Shigella* (green) injected into the hindbrain; without treatment, bacterial replication results in death of the larva. Injection of live predatory *Bdellovibrio* (red) 30–90 min after *Shigella* infection is therapeutically beneficial to the host. Here, live invasive predation of *Shigella* by *Bdellovibrio* rounds and then kills the *Shigella*, significantly reducing host bacterial burden. Remaining *Shigella* and *Bdellovibrio* themselves are ultimately cleared by host processes, including leukocyte action. Together, the immune system cooperates with predation to clear bacterial infection and promote survival.
See also Figure S3.

4. Lerner, T.R., Lovering, A.L., Bui, N.K., Uchida, K., Aizawa, S., Vollmer, W., and Sockett, R.E. (2012). Specialized peptidoglycan hydrolases sculpt the intra-bacterial niche of predatory *Bdellovibrio* and increase population fitness. PLoS Pathog. *8*, e1002524.
5. Iebba, V., Santangelo, F., Totino, V., Nicoletti, M., Gagliardi, A., De Biase, R.V., Cucchiara, S., Nencioni, L., Conte, M.P., and Schippa, S. (2013). Higher prevalence and abundance of *Bdellovibrio bacteriovorus* in the human gut of healthy subjects. PLoS ONE *8*, e61608.
6. Atterbury, R.J., Hobley, L., Till, R., Lambert, C., Capeness, M.J., Lerner, T.R., Fenton, A.K., Barrow, P., and Sockett, R.E. (2011). Effects of orally administered *Bdellovibrio bacteriovorus* on the well-being and *Salmonella* colonization of young chicks. Appl. Environ. Microbiol. *77*, 5794–5803.
7. Shanks, R.M., and Kadouri, D.E. (2014). Predatory prokaryotes wage war against eye infections. Future Microbiol. *9*, 429–432.
8. Shanks, R.M., Davra, V.R., Romanowski, E.G., Brothers, K.M., Stella, N.A., Godboley, D., and Kadouri, D.E. (2013). An eye to a kill: using predatory bacteria to control Gram-negative pathogens associated with ocular infections. PLoS ONE *8*, e66723.
9. Boileau, M.J., Clinkenbeard, K.D., and Iandolo, J.J. (2011). Assessment of *Bdellovibrio bacteriovorus* 109J killing of *Moraxella bovis* in an *in vitro* model of infectious bovine keratoconjunctivitis. Can. J. Vet. Res. *75*, 285–291.
10. Kanther, M., and Rawls, J.F. (2010). Host-microbe interactions in the developing zebrafish. Curr. Opin. Immunol. *22*, 10–19.
11. Lima, I.F., Havt, A., and Lima, A.A. (2015). Update on molecular epidemiology of *Shigella* infection. Curr. Opin. Gastroenterol. *31*, 30–37.
12. Harrington, R. (2015). Drug-resistant stomach bug. Sci. Am. *313*, 88.
13. Mostowy, S., Boucontet, L., Mazon Moya, M.J., Sirianni, A., Boudinot, P., Hollinshead, M., Cossart, P., Herbomel, P., Levraud, J.P., and Colucci-Guyon, E. (2013). The zebrafish as a new model for the *in vivo* study of *Shigella flexneri* interaction with phagocytes and bacterial autophagy. PLoS Pathog. *9*, e1003588.
14. Mazon Moya, M.J., Colucci-Guyon, E., and Mostowy, S. (2014). Use of *Shigella flexneri* to study autophagy-cytoskeleton interactions. J. Vis. Exp. *91*, e51601.
15. Schwudke, D., Linscheid, M., Strauch, E., Appel, B., Zahringer, U., Moll, H., Muller, M.z, Brecker, L., Gronow, S., and Lindner, B. (2003). The obligate predatory *Bdellovibrio bacteriovorus* possesses a neutral lipid A containing alpha-D-Mannoses that replace phosphate residues: similarities and differences between the lipid As and the lipopolysaccharides of the wild type strain *B. bacteriovorus* HD100 and its host-independent derivative HI100. J. Biol. Chem. *278*, 27502–27512.
16. Lambert, C., Chang, C.Y., Capeness, M.J., and Sockett, R.E. (2010). The first bite–profiling the predatosome in the bacterial pathogen *Bdellovibrio*. PLoS ONE *5*, e8599.
17. Karunker, I., Rotem, O., Dori-Bachash, M., Jurkevitch, E., and Sorek, R. (2013). A global transcriptional switch between the attack and growth forms of *Bdellovibrio bacteriovorus*. PLoS ONE *8*, e61850.
18. Deng, Q., and Huttenlocher, A. (2012). Leukocyte migration from a fish eye's view. J. Cell Sci. *125*, 3949–3956.
19. Renshaw, S.A., and Trede, N.S. (2012). A model 450 million years in the making: zebrafish and vertebrate immunity. Dis. Model. Mech. *5*, 38–47.
20. Hsu, K., Traver, D., Kutok, J.L., Hagen, A., Liu, T.X., Paw, B.H., Rhodes, J., Berman, J.N., Zon, L.I., Kanki, J.P., and Look, A.T. (2004). The *pu.1* promoter drives myeloid gene expression in zebrafish. Blood *104*, 1291–1297.
21. Robson, M.C., Mannari, R.J., Smith, P.D., and Payne, W.G. (1999). Maintenance of wound bacterial balance. Am. J. Surg. *178*, 399–402.
22. Zychlinsky, A., Prevost, M.C., and Sansonetti, P.J. (1992). *Shigella flexneri* induces apoptosis in infected macrophages. Nature *358*, 167–169.

Current Biology, Volume *26*

Supplemental Information

Injections of Predatory Bacteria

Work Alongside Host Immune Cells

to Treat *Shigella* Infection in Zebrafish Larvae

Alexandra R. Willis, Christopher Moore, Maria Mazon-Moya, Sina Krokowski, Carey Lambert, Robert Till, Serge Mostowy, and R. Elizabeth Sockett

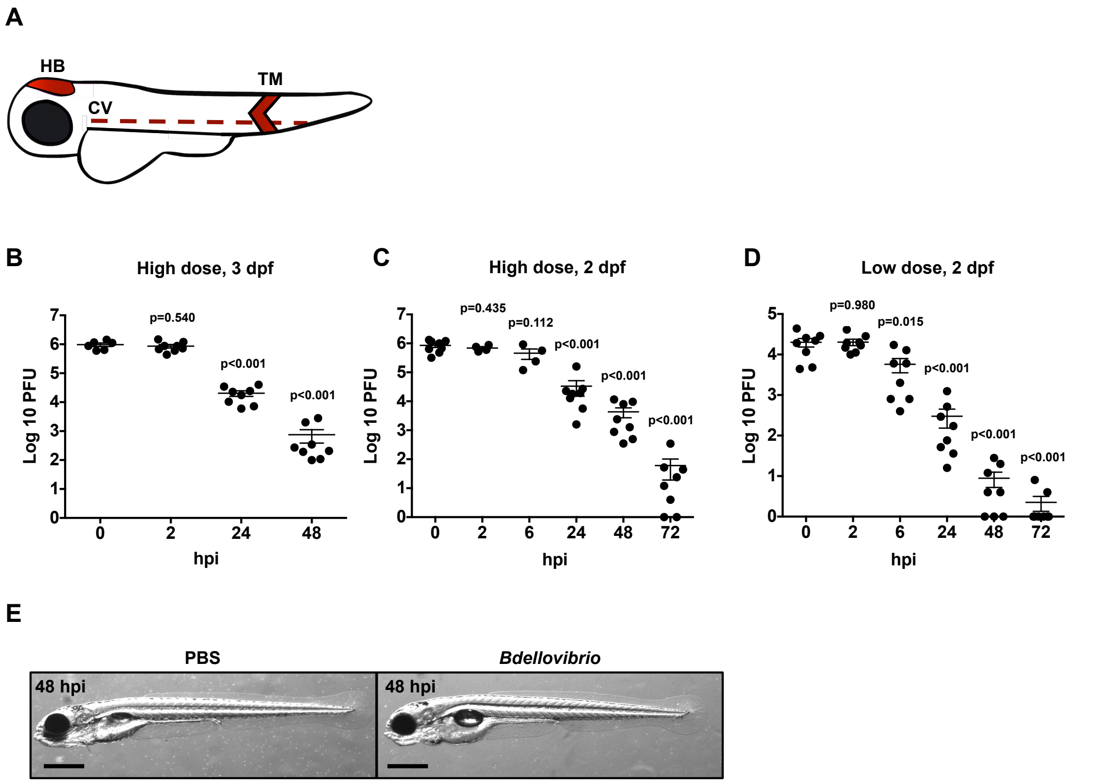

Figure S1. Injected Predatory *Bdellovibrio* Persist in Zebrafish Larvae and are Ultimately Cleared, Related to Figure 1

(A) Zebrafish larvae were injected with bacteria or PBS in either the hindbrain ventricle (HB), tail muscle (TM) or caudal vein (CV) shaded in red. Injections in the hindbrain were performed to study population level dynamics of *Bdellovibrio*, *Shigella* and/or leukocytes. Injections in the tail muscle were performed to enable high-resolution confocal microscopy.

(B–D) WT AB zebrafish larvae were injected with (B-C) a high dose of *Bdellovibrio* or (D) a low dose of *Bdellovibrio* at (B) 3 dpf or (C-D) 2 dpf in the hindbrain ventricle. Live *Bdellovibrio* were enumerated from larval homogenates at time points indicated. Each circle represents a count from an individual larva. Mean ± SEM (horizontal bars) are shown. (B) Data pooled from 2 independent experiments using 3-4 larvae per time point. (C) Data from 2 independent experiments using 4 larvae per time point. p values (versus the 0 hpi time point) determined by multiple t-test. Significance with Bonferroni correction defined as $p<0.017$ for (B), $p<0.01$ for (C-D).

(E) WT AB zebrafish larvae were injected in the hindbrain at 3 dpf with PBS or 1-2 x 10^5 PFU of *Bdellovibrio* and imaged via stereomicroscopy at 48 hpi (i.e. 5 dpf). Representative images of larval morphology are shown. Scale bar = 0.5 mm.

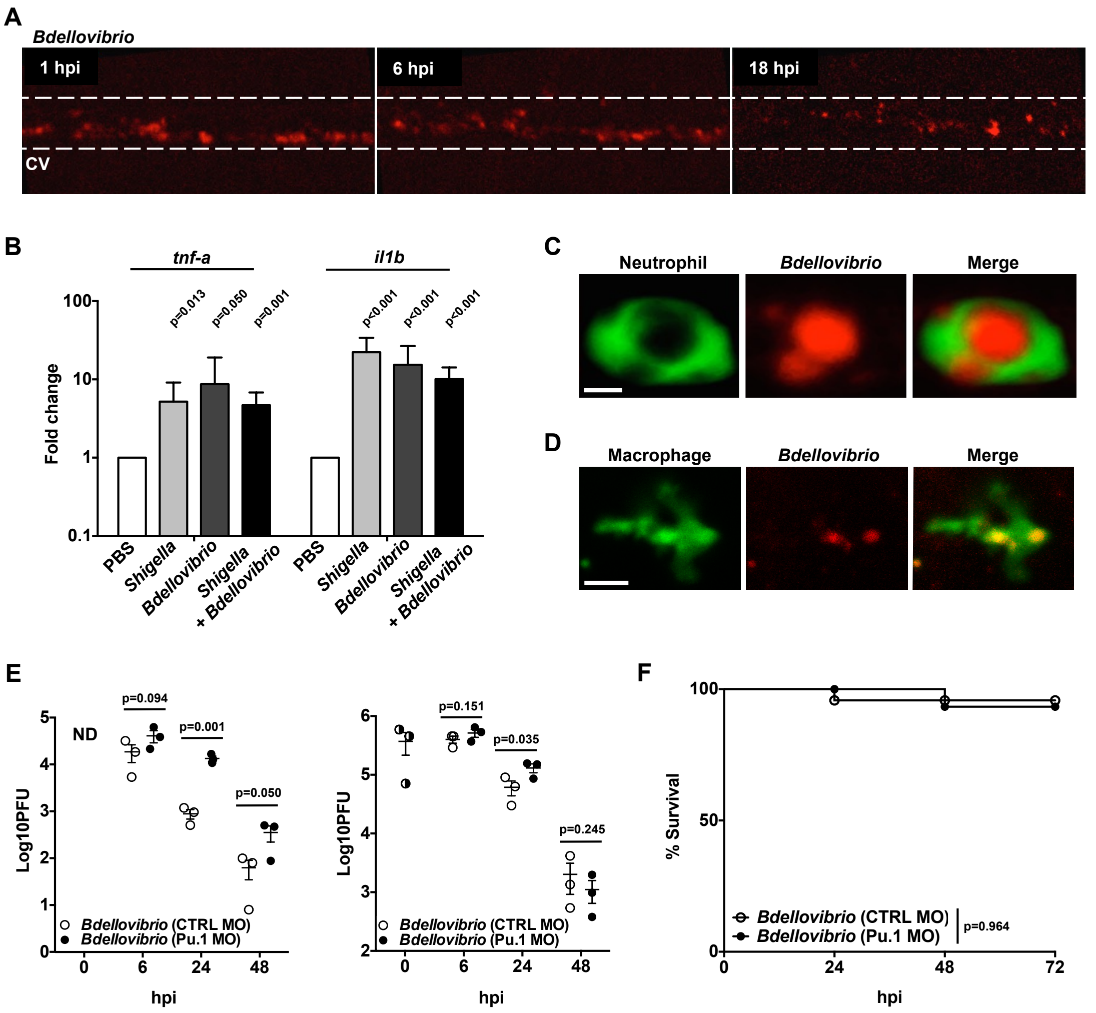

Figure S2. Characterisation of the Innate Immune Response to *Bdellovibrio* In Vivo, Related to Figure 3

(A) 1-2 x 10^5 PFU of mCherry-*Bdellovibrio* (red) were injected into the caudal vein of WT AB zebrafish larvae at 3 dpf and imaged at the stereomicroscope. The same larva was imaged over time. The area inside the white dashed line indicates the blood stream. CV, caudal vein.

(B) WT zebrafish larvae were injected with: PBS, >5 x 10^3 CFU of *S. flexneri*, 1-2 x10^5 PFU of *Bdellovibrio* or co-injected with the above doses of *S. flexneri* and *Bdellovibrio*, sequentially. RNA was extracted from pools of 5 larvae and (2 technical replicates for each of 3 biological repeats for *tnf-a*, 2 technical replicates for each of 4 biological repeats for *il1b*; normalised to PBS). Expression of *il1b* and *tnf-a* mRNA

transcripts was determined by qRT-PCR. p values (versus PBS control) determined by unpaired 1-tailed Student's t-test. Significance defined as $p<0.05$.

(C) 1-2 x 10^5 PFU of mCherry-*Bdellovibrio* were injected into the tail muscle of Tg(*mpx*:GFP)^{i114} zebrafish larvae at 3 dpf and single-cell interactions between *Bdellovibrio* (red) and neutrophils (green) visualised by high-resolution confocal microscopy at 100x magnification. A representative image is shown. Scale bar = 5 µm. See also Movie S4.

(D) 1-2 x 10^5 PFU of mTeal-*Bdellovibrio* were injected into the tail muscle of Tg(*mpeg1*:Gal4-FF)^{gl25}/Tg(UAS-E1b:nfsB.mCherry)^{c264} zebrafish larvae at 3 dpf and single-cell interactions between *Bdellovibrio* (red) and macrophages (green) visualised by high-resolution confocal microscopy at 63x magnification. A representative image is shown. Scale bar = 10 µm. See also Movie S4.

(E) Tg(*mpx*:GFP)^{i114} zebrafish larvae were pre-treated using control (CTRL) or Pu.1-targeting morpholino (MO) to deplete leukocytes. Morphants were injected at 3 dpf with either PBS or 0.1-6 x 10^5 PFU of mCherry-*Bdellovibrio*. Live *Bdellovibrio* were enumerated from PBS homogenates of larvae. Each circle represents a count from an individual larva. Half-filled circles represent enumerations from larvae at time 0 and are representative of inocula for both conditions. Mean ± SEM (horizontal bars) are shown. p values (between conditions at cognate timepoints) determined by multiple t-test. Significance with Bonferroni correction defined as $p<0.017$. As inocula from independent experiments were variable up to a log-fold (PFU counting of predators necessarily performed post experiment), representatives of 3 independent experiments performed are shown. See also Figure 3E.

(F) Survival curve of Tg(*mpx*:GFP)^{i114} zebrafish larvae injected with control (CTRL) or Pu.1-targeting morpholino (MO) and then injected at 3 dpf with either PBS or 1-2 x 10^5 PFU of mCherry-*Bdellovibrio*. Larvae were incubated at 28°C for 72 hpi. Data pooled from 2 independent experiments using n = 21-26 larvae per treatment per experiment. Up to 3 larvae per condition were taken for CFU at 24 and 48 hr

timepoints. Mean ± SEM (horizontal bars) are shown. p value between conditions determined by log rank Mantel-Cox test. Significance defined as $p<0.05$.

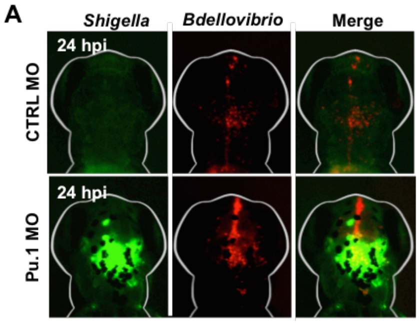

Figure S3. ***Bdellovibrio*** **Work Alongside Innate Immune Cells to Protect Against** ***Shigella*** **Infection In Vivo, Related to Figure 4**

(A) WT AB zebrafish larvae were injected with control (CTRL) or Pu.1-targeting morpholino (MO). Morphants were injected at 3 dpf in the hindbrain ventricle with > 5 x 10^3 of GFP-*S. flexneri* and treated with injection of 1-2 x 10^5 PFU of mCherry-*Bdellovibrio* 30-90 min after initial injection. Representative images of bacteria in the hindbrain ventricle in CTRL or Pu.1 morphants are shown.

SUPPLEMENTAL EXPERIMENTAL PROCEDURES

Ethics Statement

Animal experiments conducted at Imperial College London and The University of Nottingham were performed according to the Animals (Scientific Procedures) Act 1986, and were approved by the Home Office under Project Licenses: PPL 70/7446 and PPL 30/3378, respectively.

Zebrafish Husbandry

Wild type AB zebrafish were purchased from the Zebrafish International Resource Center (Eugene, OR). Tg*(mpx:GFP)*^{i114} and Tg(*mpeg1*:Gal4-FF)^{gl25}/Tg(UAS-E1b:nfsB.mCherry)^{c264} transgenic zebrafish lines are previously described [S1, S2]. Zebrafish embryos were obtained from natural spawning, bleached and raised at 28°C in E2 medium [S3]. Embryos reared for the purpose of microscopy were supplemented with 0.003% 1-phenyl-2-thiourea (Sigma-Aldrich) from 24 hours post fertilisation to prevent melanin synthesis. Zebrafish were developmentally staged according to Kimmel et al [S4]. Where specified, larvae were anaesthetised with 200 µg/ml Tricaine (Sigma-Aldrich).

Bacterial Preparations

Bacterial strains are described in the Table below. For experiments shown in Figures 1E-G, 2A, 2E-2F, 3A and S2A, non-chromosomal, cytoplasmically fluorescent *B. bacteriovorus* HD100:pK18 Bd0064mCherry (mCherry-*Bdellovibrio*) and *B. bacteriovorus* HD100:pK18 Bd0064mTeal (mTeal-*Bdellovibrio*) were prepared by predation on *E. coli*:pZMR100 prey in CaHEPES buffer (25 mM HEPES, 2 mM $CaCl_2$, pH 7.6) in the presence of 25 µg/ml kanamycin. *B. bacteriovorus* were filtered through a 0.45 µm filter to remove residual *E. coli*, washed twice with phosphate-buffered saline (PBS) to remove residual kanamycin (if present) and resuspended in PBS at 25°C. Fluorescence was eventually lost for non-chromosomally tagged

strains upon predatory replication in the absence of kanamycin selection inside zebrafish larvae. Permanently fluorescent *B. bacteriovorus* strains were constructed with chromosomal integration of Bd0064mCherry or Bd0064mTeal at the wild type gene locus and were cultured as above but in the absence of kanamycin. Permanently-tagged strains were used in all other experiments. *Bdellovibrio* suspensions were observed microscopically for purity and motility and concentrated immediately prior to injection via centrifugation. As enumerations of *Bdellovibrio* are not possible by optical density, standardised culturing was employed to give reproducible predator numbers and retrospective live cell counting of each *Bdellovibrio* culture was achieved by plaque assay counts as described in section below 'Quantification of Bacterial Burden'. Therefore, as enumeration was only available after the experiment, a small range of *Bdellovibrio* numbers may be quoted as the employed PFU.

Streptomycin- and carbenicillin- resistant *S. flexneri* serotype 5a M90T expressing green fluorescent protein (GFP-*Shigella*) was cultured overnight in trypticase soy broth (TSB) at 37°C in the presence of 50 µg/ml carbenicillin. Cultures were diluted 32 x in fresh TSB and grown until OD_{600nm} = 0.6. Bacteria were harvested via centrifugation, for 5 min at 4200 rpm, washed twice in PBS to remove carbenicillin and reconstituted in PBS.

Table. Bacterial Strains Used in This Study

Bacterial strain	Notes	Source
Shigella flexneri serotype 5a M90T-GFP	CbR, SmR	[S5]
Bdellovibrio bacteriovorus HD100:pK18 Bd0064mTeal	KanR	This work
Bdellovibrio bacteriovorus HD100:pK18 Bd0064mCherry	KanR	This work
Bdellovibrio bacteriovorus HD100 Bd0064mCherry-chromosomal	-	This work
Bdellovibrio bacteriovorus HD100 Bd0064mTeal-chromosomal	-	This work
Escherichia coli:S17-1 pZMR100	KanR	[S6]
Escherichia coli S17-1	-	[S7]

CbR, carbenicillin resistance; SmR, streptomycin resistance; KanR, kanamycin resistance

In Vitro Predation Assays

Viable counts, microscopic plate reader assays and microscopic observation of predation were carried out on *Bdellovibrio* and *Shigella* mixtures plus controls in CaHEPES buffer. For viable counting, 6.2 x 10^{10} PFU of 0.45 µm- filtered predator *bd0064*mCherry-*Bdellovibrio* and 5-12 x 10^{8} CFU of prey GFP-*Shigella* (prepared as above) were co-incubated in 10 ml CaHEPES buffer at 29°C and enumerated over time (Figure 2B). Predation from such bacterial cultures in CaHEPES was visualised via wide field microscopy (Figure 2A). Enumeration was by CFU plating for *Shigella* on Luria-Bertani (LB) agar. Enumeration of *Bdellovibrio* inoculum was by PFU plating on *E.coli* lawns on YPSC plates, as described below. For plate reader assays, 2-7 x 10^{7} CFU of GFP-*Shigella* and 8.4-10.4 x 10^{9} PFU of mCherry-*Bdellovibrio* were mixed in 200 µl CaHEPES buffer. OD_{600} and mCherry fluorescence were measured every 30 min for 6 hr at 37°C using a microplate reader (TECAN Infinite M200 Pro) but for simplicity counts were displayed every hr (Figures 2C-2D).

Morpholino Injection of Zebrafish Embryos

Antisense morpholino oligonucleotides were purchased from GeneTools (www.gene-tools.com). For leukocyte depletion experiments, published morpholinos were used to target zebrafish Pu.1, as previously described [S5, S8, S9]. To control for the effects of morpholino injection, a morpholino with no known target in the zebrafish genome was also used [S5]. Injections were performed into 1-8 cell stage embryos. Morpholinos were diluted in 0.1% phenol red (Sigma) to concentrations of 0.5-1 mM and 1-4 nl administered per embryo. Doses used were chosen to maximise leukocyte knockdown while minimising larval toxicity. Efficacy of Pu.1 morpholinos was confirmed in both Tg*(mpx:GFP)*^{i114} and Tg(*mpeg1*:Gal4-FF)^{gl25}/Tg(UAS-E1b:nfsB.mCherry)^{c26} transgenic zebrafish larvae. Larvae screened before use showed normal morphology and locomotion, an absence of macrophages and a ~50% reduction in neutrophils (typically from ~160 to ~80).

Zebrafish Infections

For hindbrain infection assays, 1-2 nl of bacterial suspension i.e. 1-2 x 10^5 PFU of *Bdellovibrio* or >5 x 10^3 CFU of *Shigella*, or PBS were delivered to the hindbrain ventricle of anaesthetised WT AB zebrafish larvae at 3 dpf via microinjection. To image single-cell interactions via confocal microscopy, tail muscle infections were performed to enable precise high-resolution imaging (Figures 2E-2F). Here, the tail muscles of zebrafish larvae were injected with 10^3 CFU of *Shigella* and 1-2 x 10^5 PFU of *Bdellovibrio*. For *Bdellovibrio* replication assays (Figure 2G), an altered ratio of predator: prey was used (i.e. 2-6 x 10^5 CFU of *Shigella* and 1-30 x 10^2 PFU of *Bdellovibrio*) to allow detection of progeny *Bdellovibrio* emerging from *Shigella* prey after replication, without being masked by a large standing number of non-invading *Bdellovibrio*. For all coinfection assays, *Bdellovibrio* was injected 30-90 min post *Shigella* infection. To test interactions with neutrophils, we used Tg*(mpx:GFP)*^{i114}

larvae with GFP-neutrophils. To study macrophages, we used Tg(*mpeg1*:Gal4-FF)^{gl25}/Tg(UAS-E1b:nfsB.mCherry)^{c26} larvae with mCherry-expressing macrophages. With the exception of Figures 2E-2F, S2A and S2C-D, all experiments including survival assays, bacterial enumerations and population level imaging via stereomicroscopy, injections were performed in the hindbrain ventricle.

Quantification of Bacterial Burden

To determine *Shigella* inoculum or burden at later timepoints, larvae were sacrificed with Tricaine, washed twice in PBS-Tricaine, lysed in 200 µl of PBS 0.1% Triton X-100 and homogenised mechanically for recovery of host-invasive *Shigella* pathogens from within the larval tissue [S5]. Here, larval homogenates were serially diluted in PBS and plated on LB agar supplemented with 50 µg/ml carbenicillin. Plates were incubated at 37°C and GFP-expressing CFU enumerated. Only viable larvae were included in our analyses.

To determine *Bdellovibrio* inoculum or burden at later timepoints, larval homogenates were prepared as described for *Shigella* above but instead homogenising in 200 µl of PBS alone as *Bdellovibrio*, unlike *Shigella*, are susceptible to Triton. PBS-treated homogenates were serially diluted and plated onto yeast extract-peptone-calcium (YPSC) soft agar overlay plates with *E. coli* S17-1 provided in the top agar to produce prey lawns for plaque formation by each individual *Bdellovibrio* [S10]. Plates were incubated for 5-10 days at 30ºC and *Bdellovibrio* enumerated as PFU.

For the co-injected *Shigella* plus *Bdellovibrio,* larval homogenates were prepared in PBS 0.1% Triton X-100. *Bdellovibrio* numbers could therefore not be co-determined as they were severely reduced by Triton treatment, required to liberate *Shigella* from host tissues.

Live Imaging, Image Processing and Analysis

For whole-animal in vivo imaging, anaesthetised zebrafish larvae were immobilised in 1% low melting point agarose and submerged in E2 supplemented with Tricaine as previously described [S11]. Visualisation of hindbrain infections was via transmission or fluorescence stereomicroscopy using a Leica M205FA microscope. Multiple position Z-stacks were acquired with a 10x (NA 0.5) dry objective, using 60 mm dishes. High-resolution imaging of tail muscle infections was via confocal microscopy using a Zeiss LSM 710 microscope. Z-stacks were acquired with a 40x, 63x or 100x oil immersion objective and subject to maximum intensity projection, using 35 mm glass-bottom dishes. Imaging was performed at 28°C. .PNG and .AVI files were processed using ImageJ/FIJI software.

Quantitative Reverse Transcription PCR (qRT-PCR) of Zebrafish Cytokines

Total RNA was extracted from pools of 5 bacterially injected or control larvae at 4 hpi using RNAqueous Kit (Ambion) and cDNA obtained using a QuantiTect reverse transcription kit (Qiagen). qRT-PCR was using SYBR green PCR master mix (Applied Biosystems) and performed on a Rotor-GeneQ thermocycler (Qiagen). Published primers were used to measure *tnf-α* and *il1b* [S12]. The housekeeping gene *gapdh* (using primers: FW-5'- TGGGCCAATGAAGGGAATTCTGGGAT-3' and RV-5'- TAACAGGTCAGCAACACGATGGCT-3') was used to normalise quantities of cDNA, using the $2^{-\Delta\Delta CT}$ method [S13]. Quantifications were performed on duplicate wells.

Statistical Analysis

All p values are displayed directly on Figures for clarity. p values for bacterial enumerations, leukocyte quantifications and cytokine data (Figures 1F, 2G, 3C-3E, 4A, S2B and S2E), determined by 1-tailed, unpaired Student's t-test on Prism

software (GraphPad Software Inc.). p values for bacterial enumerations in (Figures 1C, 2G and S1B-S1D) determined by multiple t-test on Prism software. p values for survival and in vitro assays (Figures 1G, 2B-2D, 4B and S2F) determined by log rank Mantel-Cox test on Prism software. Statistical significance is defined as p value <0.05, unless otherwise mentioned (i.e. Bonferroni correction for multiple testing).

SUPPLEMENTAL REFERENCES

S1. Ellett, F., Pase, L., Hayman, J.W., Andrianopoulos, A., and Lieschke, G.J. (2011). *mpeg1* promoter transgenes direct macrophage-lineage expression in zebrafish. Blood *117*, e49-56.

S2. Gray, C., Loynes, C.A., Whyte, M.K., Crossman, D.C., Renshaw, S.A., and Chico, T.J. (2011). Simultaneous intravital imaging of macrophage and neutrophil behaviour during inflammation using a novel transgenic zebrafish. Thromb Haemost. *105*, 811-819.

S3. Westerfield, M. (1995). The zebrafish book : a guide for the laboratory use of zebrafish (*Danio rerio*), Ed. 3. Edition, (Eugene, OR: M. Westerfield).

S4. Kimmel, C.B., Ballard, W.W., Kimmel, S.R., Ullmann, B., and Schilling, T.F. (1995). Stages of embryonic development of the zebrafish. Developmental dynamics : an official publication of the American Association of Anatomists. *203*, 253-310.

S5. Mostowy, S., Boucontet, L., Mazon Moya, M.J., Sirianni, A., Boudinot, P., Hollinshead, M., Cossart, P., Herbomel, P., Levraud, J.P., and Colucci-Guyon, E. (2013). The zebrafish as a new model for the in vivo study of *Shigella flexneri* interaction with phagocytes and bacterial autophagy. PLoS Pathog. *9*, e1003588.

S6. Rogers, M., Ekaterinaki, N., Nimmo, E., and Sherratt, D. (1986). Analysis of Tn7 transposition. Mol. Gen. Genet. *205*, 550-556.

S7. Simon, R., Priefer, U., and Puhler, A. (1983). A broad host range mobilization system for in vivo genetic-engineering - transposon mutagenesis in Gram-negative bacteria. Bio-Technol. *1*, 784-791.

S8. Clay, H., Davis, J.M., Beery, D., Huttenlocher, A., Lyons, S.E., and Ramakrishnan, L. (2007). Dichotomous role of the macrophage in early

Mycobacterium marinum infection of the zebrafish. Cell Host Microbe *2*, 29-39.

S9. Su, F., Juarez, M.A., Cooke, C.L., Lapointe, L., Shavit, J.A., Yamaoka, J.S., and Lyons, S.E. (2007). Differential regulation of primitive myelopoiesis in the zebrafish by Spi-1/Pu.1 and C/ebp1. Zebrafish *4*, 187-199.

S10. Stolp, H., and Starr, M.P. (1963). *Bdellovibrio bacteriovorus* Gen. Et Sp. N., a predatory, ectoparasitic, and bacteriolytic microorganism. Antonie van Leeuwenhoek *29*, 217-248.

S11. Mazon Moya, M.J., Colucci-Guyon, E., and Mostowy, S. (2014). Use of *Shigella flexneri* to study autophagy-cytoskeleton interactions. J. Vis. Exp. e51601.

S12. Stockhammer, O.W., Zakrzewska, A., Hegedus, Z., Spaink, H.P., and Meijer, A.H. (2009). Transcriptome profiling and functional analyses of the zebrafish embryonic innate immune response to *Salmonella* infection. J. Immunol. *182*, 5641-5653.

S13. Livak, K.J., and Schmittgen, T.D. (2001). Analysis of relative gene expression data using real-time quantitative PCR and the 2(-Delta Delta C(T)) Method. Methods *25*, 402-408.
